# Functional Group Distribution Shapes Chemical Properties of Degraded Terrestrial and Marine Dissolved Organic Matter

**DOI:** 10.1021/acs.est.5c01998

**Published:** 2025-12-01

**Authors:** Rebecca R. Matos, Alexander Craig, Boris P. Koch, Jeffrey Hawkes, Lindon W. K. Moodie, Arina Ivanova, Gerd Gleixner, Patrick Guth, Klaus-Holger Knorr, Jan Tebben, Thorsten Reemtsma, Alexander Zherebker, Oliver J. Lechtenfeld

**Affiliations:** † Department of Environmental Analytical Chemistry, Helmholtz Centre for Environmental Research − UFZ, Permoserstr. 15, Leipzig D-04318, Germany; ‡ Department of Chemistry BMC, Analytical Chemistry, 8097 Uppsala University, Uppsala 752 37, Sweden; § Department of Medicinal Chemistry, Drug Design and Discovery, Uppsala University, Uppsala 752 37, Sweden; ∥ Ecological Chemistry, 84597 Alfred-Wegener-Institut Helmholtz Zentrum für Polar- and Meeresforschung, Am Handelshafen 12, Bremerhaven 27570, Germany; ⊥ Hochschule Bremerhaven, University of Applied Sciences, An der Karlstadt 8, Bremerhaven 27568, Germany; # Department of Biogeochemical Processes, 28300 Max Planck Institute for Biogeochemistry, Hans-Knoell-Str 10, Jena 07701, Germany; ¶ Institute for Landscape Ecology, University of Münster, Heisenbergstr. 2, Munster 48149, Germany; ∇ Institute of Analytical Chemistry, University of Leipzig, Leipzig 04103, Germany; ○ Yusuf Hamied Department of Chemistry, 2152 University of Cambridge, Lensfield Road, Cambridge CB2 1EW, U.K.

**Keywords:** degradation markers, dissolved organic matter (DOM), acidity-based fractionation, sequential solid-phase extraction (SSPE), liquid chromatography Fourier transform ion cyclotron resonance mass spectrometry (LC-FT-ICR MS), proton nuclear magnetic resonance spectroscopy (^1^H-NMR), molecular tagging, deuteromethylation, functional groups

## Abstract

Dissolved organic matter (DOM) plays a crucial role in global carbon cycling, yet its molecular complexity and the factors governing its turnover and degradation in different ecosystems are poorly understood. Here, we provide an experimental assessment of structural diversity in terrestrial and marine DOM, using a multimethod approach. Terrestrial peat pore water (PPW) exhibited a similar number of COOH-groups, two times more noncarboxylic oxygen atoms (non-COOH–O, up to *n* = 20) as compared to surface seawater (SSW; up to *n* = 10), and significantly higher isomeric dispersity indices (2.5–3.0 vs 1.3–1.5), highlighting its greater structural complexity and isomeric diversity. At the level of individual molecular formulas of the widely used DOM degradation index (*I*_DEG_), we found that POS_IDEG_ molecular formulas representing fresh DOM (i.e., they were positively correlated with radiocarbon content) share similar structural characteristics in both environments (e.g., low number of carboxyl-groups). In contrast, NEG_IDEG_ markers for degraded DOM (i.e., negatively correlated with radiocarbon content) displayed a higher number of carboxyl-groups in the least acidic fraction for PPW but in the most acidic fraction for SSW. Our results indicate ecosystem-specific degradation pathways emphasizing how global carbon cycling is influenced by the molecular structure of DOM.

## Graphical abstract

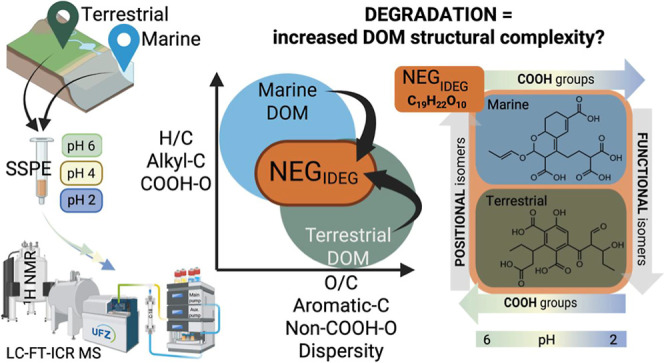

## Introduction

Dissolved organic matter (DOM) plays a major role in the global carbon cycle, contributing to both long-term storage of carbon in the oceans and terrestrial systems, which are connected via the hydrological cycle.^1–3^ Despite its importance, DOM molecular composition and thus its reactivity remains poorly understood, largely due to its molecular complexity.^4,5^ DOM consists of a complex mixture of structurally diverse compounds spanning large gradients of size, age, and biogeochemical transformations.^6^ These transformations are driven by both microbial activity and abiotic processes and are further controlled by the source of the organic material.^7^ For instance, in peatlands, the organic matter from plant litter accumulates under anoxic conditions, where it decomposes slowly via microbial processes and produces DOM enriched with highly unsaturated polyphenolic and lignin-type compounds.^8–10^ Conversely, marine DOM in the upper ocean is composed of labile algal-derived organic matter, and microbial processes further shape its composition to a more recalcitrant pool of molecules.^11–13^ A fraction of DOM in surface and deep seawater is composed of carboxyl-rich alicyclic molecules (CRAM) whereas linear terpenoid-type (MDLT) or carotenoid derived substances are likely contributing to DOM in both ecosystems.^14–17^ If analytically resolved, molecular-level changes between terrestrial and marine DOM may provide new insights into the fate of organic matter across different ecosystems.

Understanding and resolving the structural complexity of DOM remain ongoing challenges. Yet, the term “structural complexity” lacks a universal definition and varies with the molecular properties investigated. Bulk structural complexity is accessible via nuclear magnetic resonance spectroscopy (NMR),^2,4,14–17^ e.g., in combination with acidity-based sequential solid-phase extraction (SSPE).^18^ At a molecular level, the application of Fourier transform ion cyclotron resonance mass spectrometry (FT-ICR MS) in combination with derivatization techniques has provided insights into functional groups and structural motifs (i.e., the type and distribution of functional groups in individual molecular formulas (MF)).^19–21^ This approach allows differentiation between carboxylic acids (COOH-groups) and other oxygen-containing functional groups. Isomeric diversity can be characterized both statistically, based on the number of isomers (e.g., by applying the central limit theorem),^22,23^ and structurally, by analyzing chromatographic or fragmentation information.^24–27^ Here, we focus on understanding the isomeric structural composition. The combination of high-dimensional approaches (e.g., NMR, FT-ICR MS) provides a holistic perspective on the structural complexity of DOM in different environments^4,28^ and can be further refined using functional-group tagging via derivatization.^29^

Originally developed for marine systems, the degradation index (*I*_DEG_) is defined as the ratio between mass peak intensities of MF positively (POS) and negatively (NEG) correlating with bulk radiocarbon content.^30,31^ Despite the possibility that *I*_DEG_ in terrestrial environments may be affected by processes other than aging, it has been successfully applied for terrestrial DOM as an indicator of the relative degradation state of a given data set.^32–37^ Both, for terrestrial and marine DOM, *I*_DEG_ consistently increases with radiocarbon age pointing toward a generalizability of the index.^32–37^ However, despite extensive application of *I*_DEG_, no molecular level structural information for *I*_DEG_ MF has been obtained to date. This information is crucial for our understanding of how DOM degrades in marine and terrestrial settings, assuming that the *I*_DEG_ formulas are representative degradation markers in marine and terrestrial DOM.

In this study, we investigated whether structural diversity differs between two contrasting environments: a marine system represented by North Sea surface seawater (SSW) and a terrestrial system represented by peat pore water (PPW). We combined offline acidity-based SSPE followed by derivatization of COOH-groups of extracted DOM and analysis by reversed-phase (RP) liquid chromatography (LC)-FT-ICR MS. The structural interpretation of the acidity-fractionated DOM was supported by bulk ^1^H NMR and electron exchange capacity measurements. Chemical structural complexity was investigated in SSPE fractions in terms of (a) functional group distribution on a bulk level (using ^1^H NMR), (b) oxygenated functional group distribution at the individual MF level (using LC-FT-ICR MS and enumeration of COOH-groups), and (c) distribution of isomers across polarity ranges derived from RP-LC.

This new strategy was applied to the MF contributing to *I*_DEG_ (NEG_IDEG_ and POS_IDEG_) to investigate the structural differences between markers and environments. We hypothesize that NEG_IDEG_, markers of degraded DOM, exhibit a higher number of COOH-groups, although O/C ratio is similar for NEG_IDEG_ and POS_IDEG_.^30^ We also expect that degraded MF progressively converge toward a common structural composition as previously reported.^38^ The results presented here provide a novel and detailed view of DOM structural complexity by directly addressing oxygenated functionalities at the individual MF level, as well as the polarity ranges of their isomers. This methodological framework will lay the foundation to ultimately decipher DOM biogeochemical processes and carbon cycling in different ecosystems.

## Methods

### Samples

We investigated samples from two distinct regions of Germany: A peat pore water sample (PPW) from a raised bog (52°10′28.9″N 6°57′26.1″E), 42 m above sea level, dominated by terrestrial vascular plan debris, and a surface seawater sample (SSW) from the North Sea (54°08′39.0″N, 7°51′14.4″E, 25 m water depth). The pH of PPW was 4.40 and the pH of SSW was 8.22 (further details can be found in Section S1.1: Sample sites and sampling description). For a full scheme of the sample preparation, analyses and data processing refer to Section S2: Sample preparation and analysis workflow.

### Acidity-Based Sequential Solid-Phase Extraction

To fractionate DOM based on the compound’s acidity, we performed sequential extractions at three pH values: 6, 4, and 2 (Figures S1 and S2). The procedure was adapted from Zherebker et al.^39^ The SSW was adjusted to pH 6 with 30% hydrochloric acid (HCl, ultrapure; Merck) while PPW was adjusted to the same pH with 2 M sodium hydroxide (NaOH, ultrapure; Merck). No precipitation was observed for SSW and PPW. Both samples were manually extracted using styrene-divinyl-benzene sorbent cartridges (Bond Elut PPL, 5 g, Agilent, Santa Clara, U.S.A) after conditioning with 2 × 60 mL of methanol (HPLC grade, Merck) and 2 × 60 mL of pH-adjusted ultrapure water (MQW, Milli-Q Integral 5, Merck, Darmstadt, Germany) according to the manufacturer’s guidelines. The permeates were collected and acidified to pH 4 and extracted as described for pH6. Finally, the permeate from pH 4 was acidified to pH 2 and extracted. After sample loading, the cartridges were washed with 2 × 60 mL of pH-adjusted MQW and dried under N_2_. Each cartridge was eluted with 60 mL of methanol (one barrel volume) and stored in precombusted vials at −21 °C to prevent methylation.^40^ Extraction bias was limited by keeping a carbon:PPL mass ratio of approximately (0.4 ± 0.05)% (Table S1).^41^ Aliquots of fractions and permeates were dried under N_2_ flow, resuspended in acidified MQW (pH 2.0), and further analyzed for total organic carbon (TOC) (Tables S1 and S2). For the blanks, 2 L of MQW was sequentially extracted with 50 mg PPL cartridges.

### COOH-Group Derivatization Reaction

To experimentally enumerate the carboxylic acid groups, the SSPE fractions were derivatized via deuteromethylation with an adapted procedure from Zherebker et al.^42^ (Figures S1 and S2). Briefly, the volume corresponding to 1 mg of C of each methanolic fraction was dried under a N_2_ flow. Then, 120 μL of thionyl chloride (SOCl_2_, reagent grade, 97%, Merck) was added dropwise to the fraction previously redissolved in 3 mL of CD_3_OD (≥99.8 at. %D, Merck) under continuous stirring and ice-cooling. The resulting mixture was then refluxed for 6 h. Aliquots of the derivatized fractions were dried under N_2_ flow, resuspended in acidified MQW (pH 2), and further analyzed for TOC (Table S3). Afterward, the derivatized fractions were directly analyzed by LC-FT-ICR MS without further extraction.

### LC-FT-ICR MS

To separate DOM according to polarity, we used LC-FT-ICR MS as previously described.^43^ For details refer to Section S1.2: LC-FT-ICR MS and data treatment.

### Data Treatment

Chromatograms were averaged into one minute wide segments based on distinct DOM elution profiles (Figures S5 and S10) and treated as single spectra, with parameters adjusted to ensure accurate MF assignments. Derivatized samples were calibrated separately to account for deuterium incorporation, and MF assignments followed defined element constraints (for details refer to Section S1.2. LC-FT-ICR MS and data treatment and Figure S3). An isomer was defined as an MF detected multiple times over the course of the chromatographic run and across the three different pH fractions. The dispersity index was calculated as the standard deviation of retention time in which one MF was found providing a measure of polarity difference between isomers.^24^ Low dispersity index values suggest smaller structural differences within isomers causing minimal shifts in retention, as short-range variations in the position of heteroatoms or carbon branches (e.g., α, β, and γ positional changes) or carbon chain modifications with little effect on polarity. In contrast, a high dispersity index indicates larger structural differences between the isomers of the same pH fraction, likely manifesting differences in carbon skeleton, functional group composition, or position of these functional groups.

To enhance confidence and limit false assignments due to D incorporation, samples were injected in triplicate and MF subjected to general filtering and extra D and multiple assignment filters^100^ (details can be found in Section S1.2: LC-FT-ICR MS and data treatment). The maximum number of detected esters are used as an estimate of the maximum number of COOH-groups in a MF. More information regarding the calculation and limitations can be found in Section S4: Method assessment: COOH derivatization reaction. Details on the data processing workflow are found in Figures S3 and S19.

The experimental double bond equivalent to carbon ratio (DBE/C)_exp_ (eq 1) was calculated adapting the DBE/C formula. It represents the unsaturation excluding double bonds from COOH-groups, which were experimentally obtained via derivatization.^44,45^

1 $${\left(DBE/C\right)}_{\exp}=\frac{\left(non\text{‐}COOH\text{‐}C\right)+\frac{N-H}{2}+1}{\left(non\text{‐}COOH\text{‐}C\right)}$$

non-COOH–C is the total number of carbon atoms in an MF of which the number of carbon atoms in the COOH-groups was subtracted. The same way, the maximum number of non-COOH-group oxygen atoms (non-COOH–O) was calculated by subtracting the number of oxygens of a MF by the number of oxygens devoted to COOH-groups in any given MF.

### Acidity-Based SSPE and Derivatization LC-FT-ICR MS Method Assessment

The carbon mass balance during SSPE (Table S1), robustness of SSPE extracted at varying pH levels (Table S2 and Figure S4) and intermediate precision of MF intensities after LC-FT-ICR MS analysis (Table S2) were evaluated (Section S3: Method assessment: acidity-based SSPE). To understand the completeness of the derivatization reaction, the seawater DOM reference material TRM-0522 ^46^ was consecutively submitted to the derivatization reaction (two times) followed by LC-FT-ICR MS analysis (Figure S7). CRAM reference standards were derivatized following the same derivatization method (see Section S4: COOH derivatization reaction section). The derivatized CRAMs and the correspondent reference standards were then analyzed using a previously described protocol^47^ (Figure S6 and Table S4). More details are found in Section S4: Method assessment: COOH derivatization reaction. For the SSW and PPW samples, SSW exhibited a higher frequency of MFs containing COOH groups at pH 2 and 4 (Figure S8).

### ^1^H NMR

The bulk structural composition of each SSPE fraction was assessed with solution-state ^1^H NMR spectroscopy. More details are provided in Section S1.3: ^1^H NMR.

### Electrochemistry

The electron-accepting (EAC) and electron-donating (EDC) capacities of SSPE fractions were quantified using mediated electrochemical reduction (MER) and oxidation (MEO).^48^ We use EAC/EDC to quantify redox-sensitive functional moieties (e.g., quinones, but also aldehydes, and thiols) and the oxidation index (OI = EAC/[EAC + |EDC|]) to estimate the proportion of reduced versus oxidized functional groups. More details on the method and results can be found in Section S5: Electrochemistry.

### Radiocarbon ^14^C

Radiocarbon analysis was performed to obtain the overall degradation state of each fraction following a previously described protocol.^49^ More details on the method and results can be found in Section S6: Radiocarbon analysis.

## Results and Discussion

### Bulk Chemical Characterization of DOM Fractions

Surface seawater (SSW) and peat porewater (PPW) represent two distinct DOM sources, both containing mostly degraded but also labile DOM. We recovered more DOM from PPW and SSW by applying the acidity-based SSPE compared to the standard SPE method, reaching a combined yield for all three pH levels of 70%. The yields for the same samples directly extracted in a single step at pH 2 were 49% for PPW and 50% for SSW (Table S1). The applied SSPE method is robust and repeatable, resulting in clearly distinct molecular fractions (Section S3). Both terrestrial and marine DOM represent highly aged systems with bulk radiocarbon contents of 0.8541 Fm (Δ^14^ *C* = −153.4) for PPW and 0.7963 Fm (Δ^14^ *C* = −210.8) for SSW across the pH levels (Table S6). For both sources, the *I*_DEG_ values decrease with increasing pH: 0.772, 0.626, and 0.567 for PPW and 0.792, 0.607, and 0.333 for SSW for the pH levels 2, 4, and 6, respectively. Of note, *I*_DEG_ values cannot be directly transferred to other data sets, as differences in extraction procedures (here: SSPE at three pH levels) and instrumental setups (here: LC-FT-ICR MS) affect the values.^50^ Here, we use *I*_DEG_ to compare DOM eluted at different pH values (i.e., within the same environment) and not to differentiate between the two environments as both samples represent degraded DOM.

### Acidity-Based SSPE Fractionates DOM Based on Key Functional Groups and Polarity

SSPE fractions of both samples occupied distinct RT ranges in RP-LC (Figure 1A,B). The pH 6 fractions from both samples were less polar and eluted at later RT (intensity weighted retention time, RT_wa_: 18.9 min for PPW and 19.8 min for SSW) while more polar compounds were retained at pH 2 and eluted earlier (RT_wa_: 15.0 min for PPW and 16.7 min for SSW). The distinction between pH fractions was clearly observed in the compositional space, revealing a shift toward higher O/C and lower H/C ratios as pH decreased (Figure S11). Previous studies also showed that MF with high O/C and low H/C ratios are more acidic, indicating that acidity-based SSPE generally separated molecules based on their acidity/p*K*_a_ values.^39^ As for the detected *m*/*z* ranges, a similar pattern was observed across the three pH fractions of both samples, consistent with previous LC-FT-ICR MS analyses;^43,51^ both early and late retention times exhibited lower intensity weighted *m*/*z* values, whereas mid retention times tended to show higher intensity weighted *m*/*z* values­(Figure S12).

**Figure 1:**
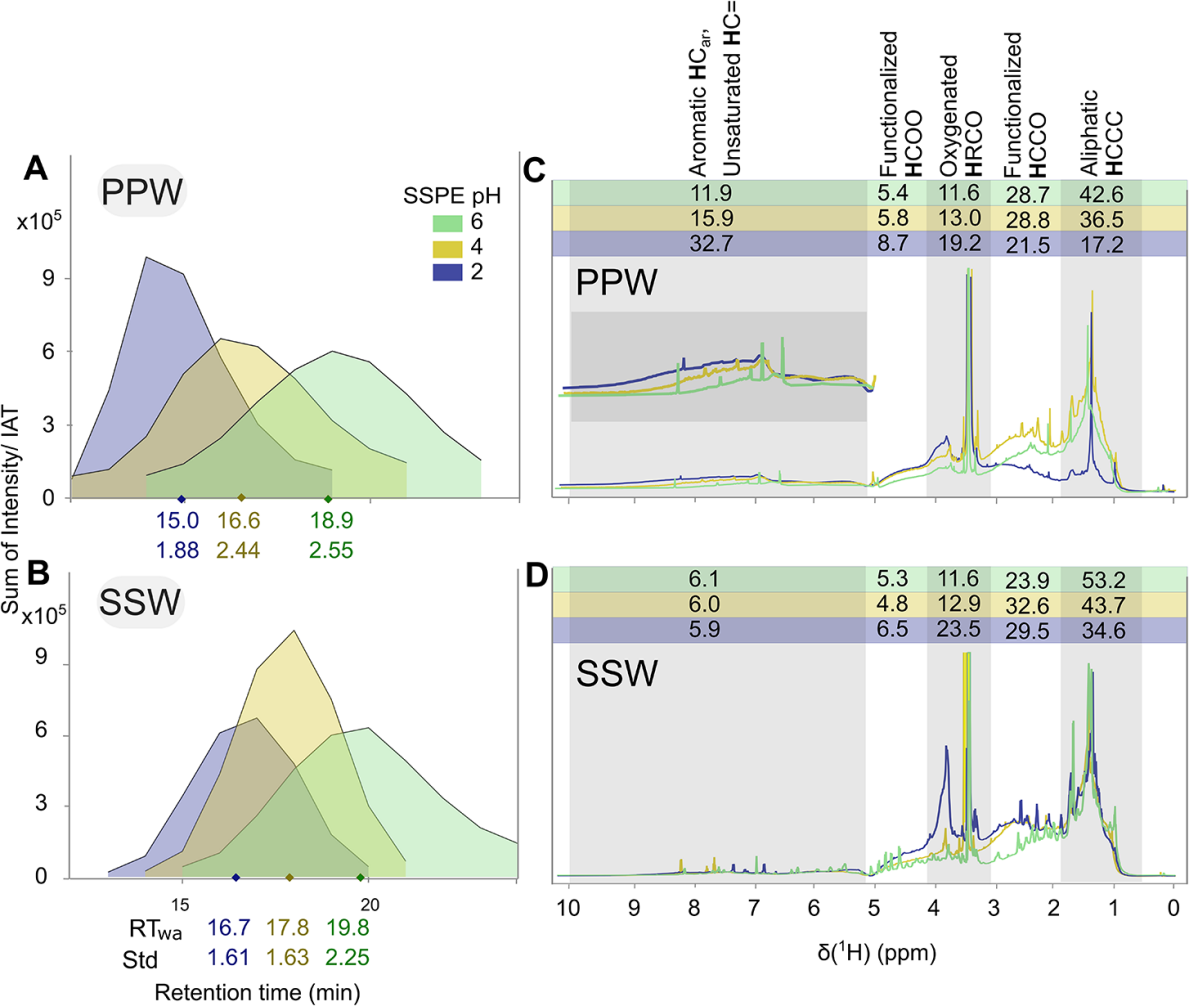
Main chemical and structural characteristics of sequential solid-phase extracted peat pore water (PPW) and surface seawater (SSW) fractions. RP LC-FT-ICR MS-derived chromatograms depicting the sum of intensity of all MF found in pH 2 (blue), pH 4 (yellow) and pH 6 (green) divided by ion accumulation time of PPW (A) and SSW (B). Weighted average retention time (RT_wa_) and its standard deviation (Std) is indicated below each chromatogram. ^1^H NMR spectra at 600 MHz in methanol-D_4_ of PPW (C) and SSW (D). The three pH fractions are superimposed using the same pH-based colors as in panel A. The gray and white boxes represent the section integrals (percent of total integral on top) as defined by key functional groups:^4,46^ Aliphatic HCCC (0.60–1.90 ppm): alkyl functionality, e.g., as polymethylene, methyl; Functionalized HCCO (1.90–3.10 ppm): two bonds away from a heteroatom, e.g., as N- and O-substituted aliphatics; oxygenated HRCO (3.10–3.20 + 3.40–4.10 ppm): one bond away from a heteroatom, e.g., as O-alkyl; functionalized HCOO (4.10–4.80 ppm): one bond away from two heteroatoms, e.g., peptide α-proton; aromatic HC_ar_ and unsaturated HC= (5.30–10.0 ppm): alkenes, aromatics; the residual methanol solvent peak is observed at 3.34 ppm.

The clear abundance shift of the major functional groups between the SSPE fractions was confirmed by ^1^H NMR spectra (Figure 1C,D). In PPW, unfunctionalized aliphatic groups (0.6–1.9 ppm) and functionalized aliphatic groups two to three bonds away from oxygen and nitrogen functionalities (1.9–3.1 pm) dominated the pH 6 fraction, while the relative proportion of aromatic/alkene groups (5.3–10 ppm) was prevalent in the pH 2 fraction, and hydrogen atoms one bond away from a heteroatom (3.1–4.1 ppm) also increased. This aligns with the trend observed in PPW’s O/C and H/C ratios (Figure S11A), confirming a shift from aliphatic to aromatic substructures (evidenced by a decrease in the H/C ratio from pH 6 to pH 2) and an increase in oxygen-containing functional groups (reflected in the corresponding rise in the O/C ratio). Acidity-based SSPE performed on Leonardite humic material also separated aliphatic and aromatic components with aliphatic functional groups dominating at higher pH.^18^ High EAC and EDC values were observed for PPW across all pH fractions, indicating a large number of functional groups that can be reduced or oxidized within the range of naturally occurring redox conditions (Eh: +0.68 to −0.36 V), such as quinones and partially phenols and aldehydes (Table S5). The values are within reported values for peat systems.^10,52^

Although the ^1^H NMR spectra for SSW did not indicate an increase in aromatic/alkene content from the pH 6 to 2 fraction (Figure 1D), the (H/C)_wa_ decreased with decreasing pH from 1.45 to 1.28 (Figure S11B). Therefore, double bond equivalents in SSW must be attributed to other functional groups, such as carbonyl groups (where CHx or OH is replaced by CO/COOH), ring formation (e.g., hexane to cyclohexane), or fully substituted alkenes or aromatics. Lower EAC/EDC values in SSW as compared to PPW suggest functional groups that are redox-insensitive within the Eh range covered, e.g., carboxylic acids.^53^ The consistently lower oxidation index confirms a larger proportion of reduced functional groups in PPW compared to the highly oxidized SSW (Table S5).

Overall, stark differences in molecular composition and key functional groups confirmed that acidity-based SSPE separated DOM into chemically distinct fractions. At the most alkaline fraction (pH 6), both samples were rich in alkyl substructures. The more acidic fractions (pH 4 and 2) revealed that PPW was dominated by aromatic substructures (i.e., quinones and phenols, typically for lignin- and tannin-derived compounds) with low content of aliphatic protons, while SSW was enriched with carbonyl and nonaromatic rings contributing to molecule unsaturation (as found in e.g., material derived from linear terpenoids, MDLT, and CRAM).

### Higher Diversity of Oxygen-Containing Functional Groups in Terrestrial than in Marine DOM

While the overall aggregated molecular information from ^1^H NMR and polarity separation via LC-FT-ICR MS provides a useful overview of the distribution of key functional groups, the derivatization of the COOH-groups revealed structural differences in molecules with identical MF across different fractions. COOH-group derivatization was performed experimentally through a reaction with deuterated methanol in each of the SSPE fractions and analyzed using LC-FT-ICR MS without further extraction. Consequently, elemental composition of PPW and SSW constituents was refined by structural features: the estimated number of COOH-groups and the number of oxygen and carbon atoms not bound as COOH-groups (i.e., non-COOH–O and non-COOH–C). Control labeling studies using model CRAM compounds highlighted that complete labeling was observed for triacid compounds, but only three out of a possible four OCD_3_ labels were incorporated for tetra-acid standards with a 1,1, diacid functionality (Section S4). As such, it is possible that labeling of SSPE fractions slightly underestimated the upper limit of total carboxylic acid functionalities for any given isomer. Notably, for all model compounds formulas representing one and two OCD_3_ labels were observed. Such series of labeled MF within SSPE extracts may represent multiple derivatives of one isomer, as anticipated.^54^ However, this feature of the data was incorporated into our interpretation, which only considers the highest number of labels and is therefore unlikely to affect the interpretation of differences between SSPE samples, as incomplete labeling in this manner will occur in all extracts. Derivatization of COOH-groups thus provides reasonable estimates of the number of COOH groups in terrestrial and marine molecules.

Non-COOH–O accounted for twice as many oxygen atoms in PPW compared to SSW (up to 20 in PPW, Figure 2A). This is further supported by the higher EEC values in PPW, indicating the presence of redox-sensitive oxygen functionalities such as quinones, aldehydes, and phenols (Table S5). In the pH 6 fraction of PPW, a high number of COOH-groups was associated with fewer non-COOH–O and low unsaturations (average DBE/*C*_exp_ value of 0.4), indicating that these COOH-groups are attached to saturated alkyl chains. This is further supported by ^1^H NMR data, which showed a high proportion of alkyl functionalities at pH 6 (Figure 1C).

**Figure 2:**
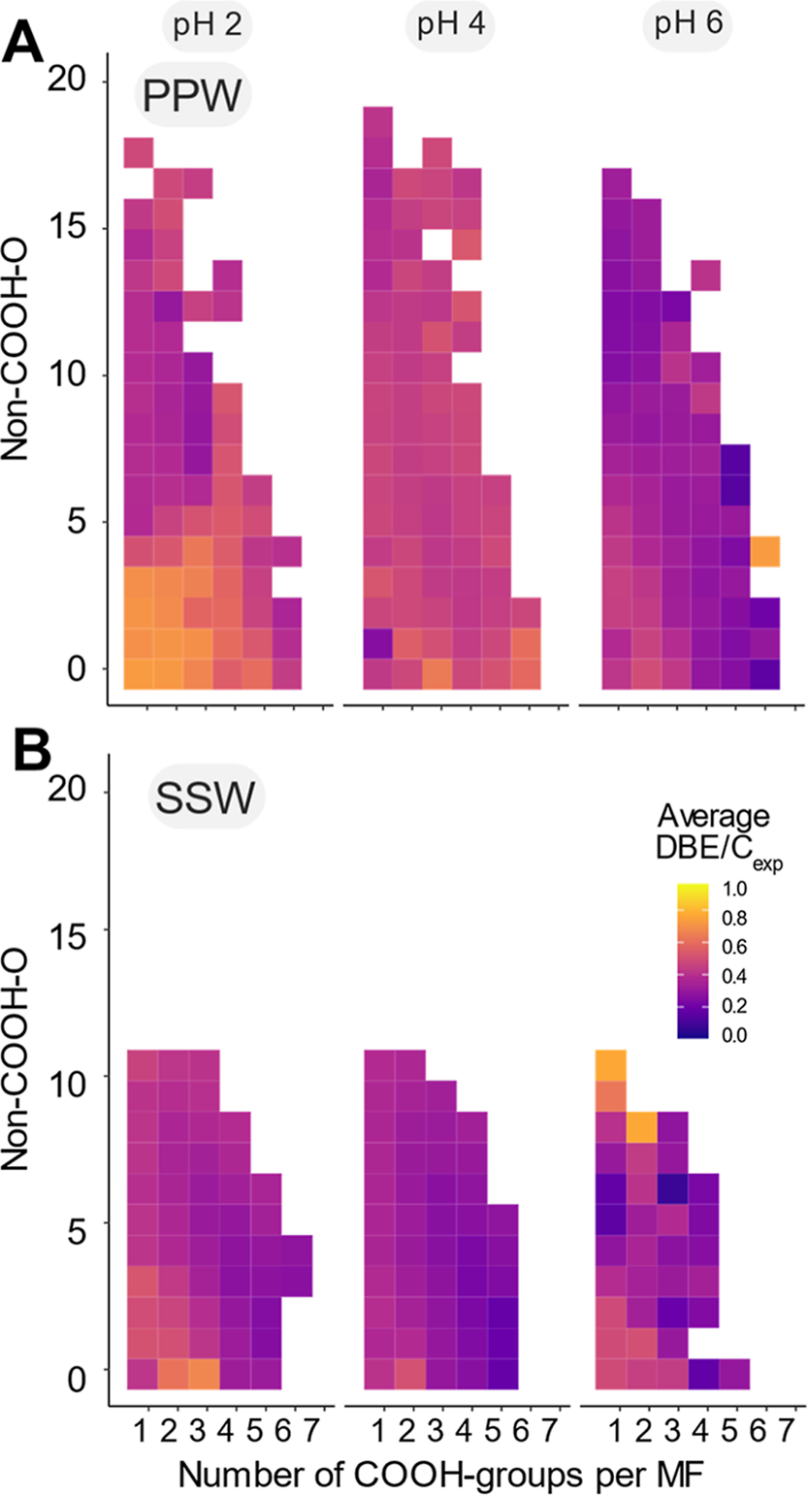
Distribution of carboxylic acid groups and oxygen atoms not bound as carboxylic acid groups. The number of carboxylic acid groups (COOH-groups) was obtained via derivatization of acidity-based sequential solid-phase extracted DOM from (A) peat pore water (PPW) and (B) surface seawater (SSW). The number of non-COOH oxygen (non-COOH-O) atoms was calculated from the respective MF. Each bin is colored by the average experimental double bond equivalent to carbon ratio (DBE/*C*_exp_, cf. method section) of the underlying MF. White bins indicate the absence of MF in that category. The number of MF per bin ranged from 1 to 890. Higher DBE/*C*_exp_ values in PPW compared to SSW and the increase in DBE/*C*_exp_ with decreasing SSPE pH suggest a greater contribution of aromatic and unsaturated compounds in PPW, particularly for the more acidic fractions.

As the pH decreased, unsaturation increased (average PPW DBE/*C*_exp_ at pH 4 of 0.5 and at pH 2 of 0.6, Figures 2A, S13 and S14) while the number of COOH-groups per MF remained consistent across pH levels (ranging from 0 to 6). Highly unsaturated molecules with a high number of COOH-groups suggest that COOH-groups are more likely directly linked to aromatic systems. There is a consistent difference in molecule unsaturation between sources, with PPW having higher DBE/*C*_exp_ values than SSW, yet both samples share the same MS-derived mass range. This suggests that the low DBE/*C*_exp_ in SSW reflects the absence of aromatic systems rather than an artifact of a lower carbon number (Figure S15). Given that the DBE/*C*_exp_ values found for PPW at pH 4 and 2 were higher than those typically associated with one aromatic ring (DBE/C ≥ 0.5)^44^ and that the number of non-COOH–O was also high, it is likely that PPW carried other oxygenated functions bound to both sp^2^ carbons (phenols, ketones, esters, and aldehydes, typically associated with polyphenols and lignin degradation products) and partly also sp^3^ carbons (alcohols and ethers). This aligns with the observation from RP retention times of oxygen-functionalized CRAM model compounds and chemical databases, suggesting that alcohols are important functional groups driving molecule polarity.^55,56^ COOH-groups are (mostly) protonated at pH-values typically used in RP-LC (here: pH 3) facilitating retention. In contrast, polyols (e.g., sugars) are known to have poor SPE recovery at pH 2 and d-glucuronic acid (log*D* = −3.7) used in previous applications of our LC-method showed a low RP-LC retention at this pH. A higher contribution of alcohols can explain the lower RT_wa_ values of PPW as compared to SSW at same extraction pH values (Figure 1A,B).^43,57^ Overall, in terrestrial DOM, highly carboxylic acid-functionalized molecules tend to be aliphatic and with lower RP-LC-based polarity, while the more acidic and highly polar DOM fractions revealed increasingly unsaturated and aromatic molecules with more variable oxygen functional group compositions.

For the SSW fractions, DBE/*C*_exp_ decreased with an increasing number of COOH-groups in the more acidic fractions (pH 2 and 4, from DBE/*C*_exp_ = 0.50 and 0 COOH-groups to DBE/*C*_exp_ = 0.32 and up to 6 COOH-groups at pH 2, Figure S14). Particularly for higher numbers of COOH-groups per MF and smaller molecules, the double bonds are more likely to be confined to the C=O bond of the COOH-group (Figures 2B and S16B). As a result, the remaining oxygen atoms were more likely bound to sp^3^ carbons as alcohols or ethers, confirmed by ^1^H NMR (Figure 1D). Consequently, an increasing number of COOH-groups leads to a narrower range of possible functional group distributions, which is reflected in the low mean and range of DBE/*C*_exp_ values (Figures S13 and S14). Our results for SSW are in good agreement with the proposed structural features of CRAM.^2,46^ Notably, here, we provide experimental evidence for CRAM structural features of individual molecules using derivatization combined with LC-FT-ICR MS.

In contrast to PPW, the COOH-groups accounted for most of the unsaturation in SSW, confining other oxygenated functionalities bound to sp^3^-carbons. Overall, our results show that the variability of oxygen-containing functional groups is lower in marine compared to terrestrial DOM, confirming a transition from functionally diverse, aromatic-rich terrestrial DOM to less functionally complex, alkyl-dominated marine DOM.

### Higher Isomeric Complexity in Terrestrial than in Marine DOM

We used the dispersity index as a measure of the retention (i.e., polarity) difference between structural isomers in RP-LC.^24^ While a high dispersity index can be associated with a large isomeric complexity, a low dispersity index reflects more similar structures.

Dispersity index distributions revealed pronounced differences between the samples and pH fractions. SSW covered a narrower RT range per MF within the most acidic fractions (pH 2 and 4) when compared to PPW (Figure 3). The dispersity index modes (i.e., the most frequently observed dispersity index values) were 1.30 and 1.48 for SSW at pH 2 and 4, respectively, but 2.45 and 3.02 for the same PPW fractions. This suggests that within SSW fractions, isomers most likely represented more similar chemical structures as compared to PPW, eluting in a narrow polarity range (i.e., within few 1 min segments). The number of COOH-groups only had a minor effect on dispersity (Figure S17B), implying that isomers in SSW reflect a more constrained core structure. Positional isomers (same carbon chain structure but differing position of functional groups) or chain isomers (differing arrangement of the carbon skeleton while retaining type and number of oxygen functional groups) likely prevailed in the acidic fractions of SSW (pH 2 and 4), suggesting that next to the type of functional group, their position in relation to the carbon core impacts the polarity differences (i.e., dispersity values).

**Figure 3:**
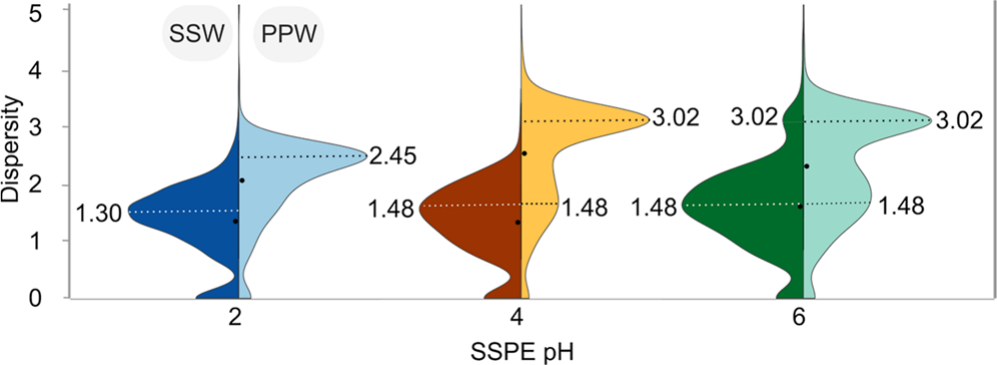
Isomeric complexity in peat pore water (PPW) and surface seawater (SSW) as a function of extraction pH. Dispersity index distribution of SSW (left, darker colors) and PPW (right, light colors) for SSPE at pH 2 (blue), pH 4 (yellow), and pH 6 (green). The dotted lines indicate the modes, i.e., most repeated values of dispersity indices.

Two distinct groups of molecules (cf. two modes in Figures 3 and S18B) were observed in PPW at pH 4 and 6, while SSW showed just one mode across all pH fractions. High dispersity indices (between 2.5 and 4) dominated in PPW for MF with high DBE-O values, between −3 and 5, while MF with DBE-O values <−3 corresponded to lower dispersity index values (<2.5) (Figure S19B). This indicates that an increasing number of double bonds and/or a low number of oxygens in a MF results in striking structural differences that affect the polarity distribution of the isomers in PPW (Figure S18A). The broad dispersity range observed for the same number of COOH-groups in PPW (Figure S17) suggests that isomers were not limited by a core structure but rather were influenced by the substantial variability of non-COOH oxygen functionalities, supporting the evaluation of the oxygen functionalities above. The dispersity index increased for MF with up to 3 COOH-groups and decreased again with higher number of COOH-groups. In the most alkaline fraction (pH 6) of both samples, the dispersity index increased to higher values (>2.5). The high content of alkyl functionalities (Figure 1C,D) found at pH 6 supported a high number of isomers with a large RT range.

Previous studies have suggested that a high dispersity and similar fragmentation spectra (due to chimeric MS/MS averaging) indicate large structural diversity.^22,23,26,38,58^ Our results allow us to differentiate functional group isomers from positional/chain isomers, showing that both types are more pronounced in terrestrial than in marine DOM. While our approach can approximate the type and chemical characteristics of isomers, it still cannot determine the exact number of isomers. Even a small number of functional group types, such as in CRAM, can lead to a large number of potential isomers,^14^ which share similar chemical properties (e.g., polarity) and reactivity (e.g., bioavailability).

Overall, isomeric complexity transitions from structurally diverse, likely functional isomers in PPW to simpler, more uniform positional and chain isomers in SSW. The high dispersity indices in PPW were linked to greater structural complexity, as isomers display significant differences in polarity, driven by an increased number of double bonds and non-COOH–O functionalities. This suggests that PPW isomers exhibit a high degree of complexity due to the diversity of oxygenated functional groups and larger differences in the core structure. In contrast, SSW shows lower dispersity, with isomers, limited by a more confined core structure, covering a narrower polarity range, where COOH-groups and other oxygenated functionalities are balanced.

### NEG_IDEG_ MF have a Higher Number of COOH-Groups than POS_IDEG_

Our multimethod approach using SSPE, derivatization, and LC-FT-ICR MS allows us to pinpoint structural groups and isomeric complexity of individual oxygenated compounds within complex DOM. We chose an exemplary MF (C_19_H_22_O_10_ with O/C = 0.52, H/C = 1.16, DBE = 9), which represents a marker for degraded DOM with even number of oxygens atoms (i.e., a NEG_IDEG_ MF that increases in relative abundance with age),^30^ to depict possible chemical structures of isomers in terrestrial and marine DOM (Figure 4). Here, the co-occurrence of the same MF in multiple 1 min segments at the same or different pH fractions was treated as putative distinct isomers. This still represents a lower limit since we expect that isomers coelute during a 1 min RT segment (Figure S6).

**Figure 4:**
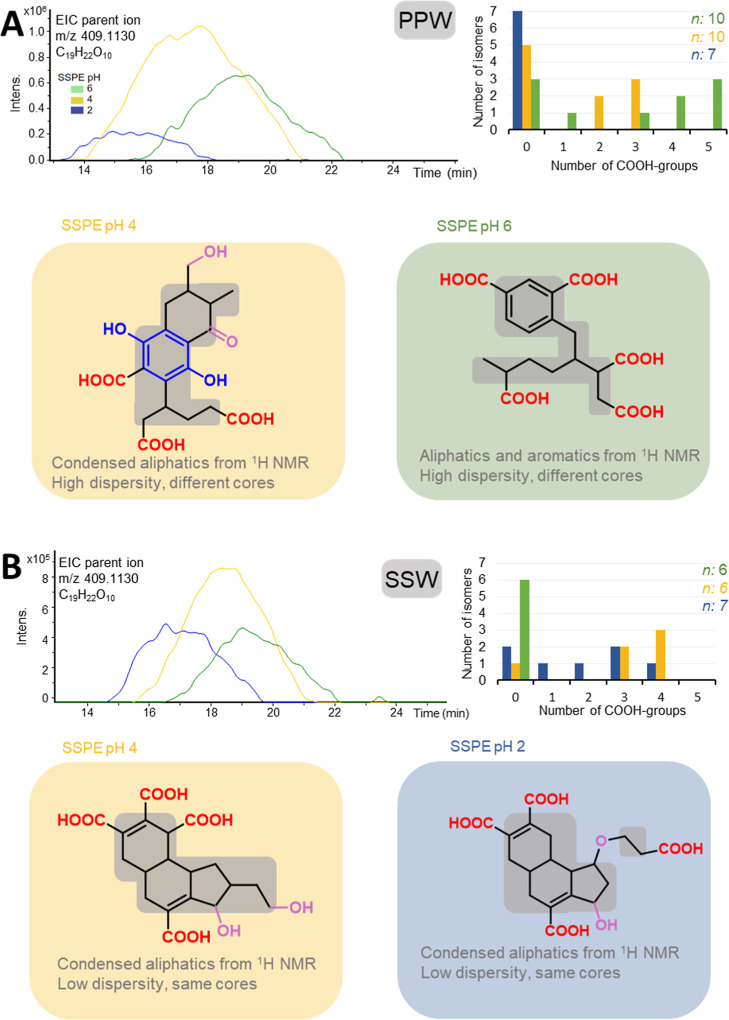
Proposed chemical structures for the NEG_IDEG_ MF C_19_H_22_O_10_ (*m*/*z* 409.1130). Extracted ion chromatograms (EICs, top left) are shown for (A) peat pore water (PPW) and (B) surface seawater (SSW) extracted at pH 6 (green), 4 (yellow), and 2 (blue). For each sample and pH, the maximum number of carboxylic acid groups detected for each isomer (for details about the counting of isomers, refer to Figure S9) is shown in the top right. The total number of isomers (i.e., retention time segments as lower estimate of isomers) in which the MF was found at each pH is indicated by “*n*”. Below, chemical structures for C_19_H_22_O_10_ suggested from multiple analytical techniques: The number of COOH-groups (highlighted in red on the structures) was derived from the derivatization (cf. Figure 2, with isomer containing the highest number of COOH-groups shown (PPW pH 4:3; PPW pH 6:5; SSW pH 4 and pH 2:4). Indications from electrochemical data are overlaid in blue, and values for DBE and O balance after COOH-derivatization are shown in purple (cf. Figure 2). Core structural motifs (gray boxes) were proposed based on ^1^H NMR data and dispersity index analysis (cf. Figures 1 and 3). Note that the exact positions of functional groups and the distribution of O atoms to non-COOH groups could not be determined.

A high number of COOH-groups per isomer of C_19_H_22_O_10_ was observed for the least acidic fraction in PPW (up to 3 isomers with 5 COOH-groups at pH 6), while SSW had the highest number of COOH-groups per isomer in the most acidic fraction (up to 1 and 2 isomers with 4 COOH-groups at pH 2 and 4). The high dispersity indices for C_19_H_22_O_10_ in PPW (3.02 at pH 4 and 6 and 2.16 at pH 2) suggested significant changes in the structure and distribution of functional groups of the underlying isomers, likely contributing to its broad chromatographic elution (Figure 4A). In addition to its high number of double bond equivalents (DBE/C = 0.5), C_19_H_22_O_10_ likely contained a mix of sp^2^ and sp^3^ hybridized carbons bound to oxygens, reflecting its greater isomeric structural diversity. Notably, at pH 2, we did not detect COOH-groups for this MF in PPW (Figure S19), indicating that the isomers in the most acidic fraction were driven by polyphenolic or other oxygen-containing aromatic compounds. It must be noted that only compounds not extracted at pH 6 and pH 4 can be expected in the pH 2 extract. Our results also suggest that COOH-rich compounds from PPW were less acidic as their SSW counterparts (i.e., are already retained at higher pH) and that only the most polar, polyphenols/polyols remain in the pH 2 extract. Absence of COOH-groups is a consistent pattern also observed for most POS_IDEG_ MF at pH 2 (Figure S19).

In contrast, the lower dispersity indices for C_19_H_22_O_10_ in SSW (2.16 at pH 2 and 6 and 1.17 at pH 4) suggest that isomers of this NEG_IDEG_ MF have minimal variations in the types or positions of functional groups which consequently did not cause significant shifts in retention time, particularly evident at pH 4 (Figure 4B). In the acidic fractions (pH 2 and 4), double bonds were confined to COOH-groups (up to 4 COOH groups), carbon–carbon double bonds, and rings, while the remaining non-COOH–O atoms were likely bound to sp^3^ hybridized carbons, forming alcohols and ethers (see section Higher diversity of oxygen-containing functional groups in terrestrial than in marine DOM). Of note, the differences in structures of isomers will also have an implication for its mass spectrometric ionization and detection^59^ as well as fragmentation pattern^26,57^ as can be assumed for the pH fractions with and without COOH-groups.

The other MF contributing to NEG_IDEG_ showed similar chromatographic behavior and functional group distribution pattern as C_19_H_22_O_10_, i.e., a high number of COOH-groups in acidic fractions for SSW but more alkaline fractions for PPW (Figure S18), suggesting that they represent biogeochemically similar markers. NEG_IDEG_ MF have been shown to accumulate over time and with advancing degradation as compared to POS_IDEG_ MF.^30,31,36^ A high proportion of COOH-groups for NEG_IDEG_ MF indicates progressive oxidation, as expected for highly recalcitrant, degradation-resistant molecules.^30,31,36,47^

For both, terrestrial and marine DOM, NEG_IDEG_ MF were associated with a higher number of COOH-groups as compared to POS_IDEG_ MF. Since POS_IDEG_ MF are removed as degradation progresses,^30^ the lability of POS_IDEG_ MF can thus be linked to the lack of carboxylic acids groups in the individual molecules. Our findings align with a recent work suggesting that CRAM-like DOM increase in relative abundance as DOM undergoes degradation in aquatic environments.^43,51^

### NEG_IDEG_ Are Structurally Diverse while POS_IDEG_ Are Uniform in Terrestrial and Marine DOM

The different dispersity indices for the NEG_IDEG_ MFs between terrestrial and marine DOM highlight the contrasting structural complexity and variability of isomers in each environment. While PPW shows greater isomeric diversity due to molecule unsaturation and a variety of oxygenated functionalities, SSW is characterized by a less diverse core structure and oxygen-bearing groups, leading to a more uniform chromatographic behavior. For the first time, our experiments provide structural information at the individual MF level about DOM degradation markers, highlighting the stark differences on the structural level. This observation calls for a cautionary use of *I*_DEG_ when comparing diverse ecosystems. While *I*_DEG_ has been found to provide robust estimates on the *degradation state* of DOM across environments,^30,35^ it may not indicate similar *degradation pathways*.

In contrast to NEG_IDEG_, the molecular marker for fresh DOM (i.e., POS_IDEG_ MF, Figure S18) exhibits a more uniform functionalization (with overall low number of carboxylic acids estimated per isomer; ≤3 COOH-groups), similar polarity (RT_wa_) across pH fractions, and hence presumably more similar structures in both marine and terrestrial environments. Although both samples from this study represent extensively degraded DOM (Table S6), individual DOM compounds are expected to represent a continuum of degradation states.^30,31^ POS_IDEG_ markers indicate younger, more labile DOM that is consumed over time, while NEG_IDEG_ represents signatures of reworked and more degraded organic matter. The structural differences between POS_IDEG_ and NEG_IDEG_ support the idea of increasing molecular richness during degradation and mixing.^60^ However, the distinct chemical structures of NEG_IDEG_ in marine versus terrestrial DOM suggest contrasting degradation pathways and an accumulation of site-specific DOM compounds as degradation advances contrasting earlier observations of a decrease in site-specific DOM.^38^

In summary, the high COOH-group content and large structural diversity of NEG_IDEG_ MF reaffirm their recalcitrant nature, particularly in terrestrial environments. POS_IDEG_ MF, on the other hand, reflects more labile, structurally less diverse components that degrade more readily in both marine and terrestrial environments, though their exact chemical structures and degradation pathways may vary across these systems.

### Biogeochemical Implications

This study utilizes advanced analytical methods (radiocarbon dating, electrochemical oxidation/reduction, NMR, and LC-FT-ICR MS) and functional group derivatization to experimentally assess the structural complexity of two DOM samples representing terrestrial and marine environments at the level of isomers. The methodology advances the analysis of DOM chemical space beyond MF and incorporates direct structural information about oxygen-containing functional groups as well as about unsaturation for chemically distinct isomeric groups. Our results based on individual MF highlight that each MF represents a multitude of oxygen-containing functional groups and isomers with structures differing strongly between terrestrial and marine DOM. Oxygen-containing functional groups are crucial to understand DOM cycling and degradation, as they are involved in manifold biological (e.g., bioavailability, metabolic energy yield) and abiotic reactions (e.g., aquatic mobility and adsorption to surfaces).

We showed that terrestrial DOM is more susceptible to changing redox conditions (higher number of non-COOH–O), resembling signatures of its unique structural sources (high aromatic/phenolic content), even for the most degraded fraction. Apparent low COOH content of the most acidic fractions in PPW suggests that solubility and mobility are at least partially controlled by aromatic units and polyols suggesting cosolubilization of molecules in high DOM concentration systems.

Marine DOM showed fewer functional group types but more oxygen atoms and double bonds associated with carboxylic acids. We can, for the first time, structurally address CRAM-type molecules at the individual MF level. A lower isomeric complexity in marine compared to terrestrial DOM was manifested by lower retention time variability, suggesting that degradation in marine systems favors a more uniform and chemically similar DOM pool, which, however, exists at minute concentrations.

A high number of COOH-groups in NEG_IDEG_ markers confirms that oxidation of DOM is a major driver for its persistence with, again, stark differences between environments. Surprisingly, the occurrence of COOH-rich NEG_IDEG_ isomers in PPW in higher pH fractions than isomers with lower number of COOH suggests that this fraction of terrestrial DOM may be lost during land-ocean transfer (e.g., low solubility at high ionic strength, photolysis) or that initially polar, polyphenol-like compounds are transformed via oxidative dearomatization and oxidation of non-COOH–C resulting in CRAM-like compounds with consequently higher acidity (and RP-LC retention at pH 3).^15^ The structural divergence between POS_IDEG_—simpler, more uniform chemical structures with fewer COOH-groups—and NEG_IDEG_ markers can be utilized to better understand and experimentally assess the distinct geochemical processes governing DOM turnover in marine and terrestrial ecosystems.

## Supplementary Material



## Data Availability

Processed and quality-checked data for all samples and segments of LC-FT-ICR MS of SSPE and derivatized extracts, as well as 1H NMR of SSPE extracts and CRAM LC-Orbitrap MS before and after derivatization, are available from the UFZ Data Investigation Portal: 10.48758/ufz.16221. Raw MS files can be shared upon request.

## Notes

Additional information regarding sample collection, preparation, and analysis performed, data processing workflow, DOC determination, SSPE method assessment, COOH-group derivatization method assessment, CRAM reference material chromatograms, LC TIC chromatograms, electrochemistry data, radiocarbon data, Van Krevelen diagrams of SSPE extracts, new calculated descriptors of derivatized data, and IDEG LC EIC chromatograms (PDF)
