# Supplementary material for: Functional Group Distribution Shapes Chemical Properties of Degraded Terrestrial and Marine Dissolved Organic Matter
Source: Environ Sci Technol. 2025 Dec 1;59(49):26539–49. doi: 10.1021/acs.est.5c01998 (PMC12713765; doi:10.1021/acs.est.5c01998)
Supplement: Supplementary file 1 [file es5c01998_si_001.pdf]

Supplemental Information

Functional Group Distribution Shapes Chemical Properties of Degraded Terrestrial and Marine Dissolved Organic Matter

Rebecca R. Matos[1]*, Alexander Craig[2,3], Boris P. Koch[4,5], Jeffrey Hawkes[2], Lindon W. K. Moodie[3], Arina Ivanova[6], Gerd Gleixner[6], Patrick Guth [7], Klaus-Holger Knorr[7], Jan Tebben[4], Thorsten Reemtsma[1,8], Alexander Zherebker[9], Oliver J. Lechtenfeld[1]*

[1] Department of Environmental Analytical Chemistry, Helmholtz Centre for Environmental Research − UFZ, Permoserstr. 15, Leipzig D-04318, Germany

[2] Department of Chemistry BMC, Analytical Chemistry, Uppsala University, Uppsala 752 37, Sweden

[3] Department of Medicinal Chemistry, Drug Design and Discovery, Uppsala University, Uppsala 752 37, Sweden

[4] Ecological Chemistry, Alfred-Wegener-Institut Helmholtz Zentrum für Polar- and Meeresforschung, Am Handelshafen 12, Bremerhaven 27570, Germany

[5] Hochschule Bremerhaven, University of Applied Sciences, An der Karlstadt 8, Bremerhaven 27568, Germany

[6] Department of Biogeochemical Processes, Max Planck Institute for Biogeochemistry, Hans-Knoell-Str 10, Jena 07701, Germany

[7] Institute for Landscape Ecology, University of Münster, Heisenbergstr. 2, Münster 48149, Germany

[8] Institute of Analytical Chemistry, University of Leipzig, Leipzig 04103, Germany

[9] Yusuf Hamied Department of Chemistry, University of Cambridge, Lensfield Road, Cambridge CB2 1EW, United Kingdom

* Corresponding Author: rebecca.matos@ufz.de; oliver.lechtenfeld@ufz.de

Contents

1. Method details 6

1.1. Sample sites and sampling description 6

1.2. LC-FT-ICR MS and data treatment 6

1.3. ^{1}H NMR 7

2. Sample preparation and analysis workflow 8

Figure SI 1 – Sample location and sample preparation procedure (acidity-based SSPE and derivatization). Peat pore water (PPW) and surface sea water (SSW) samples were obtained in Germany (as described in "**SI Section 1: Sample sites and sampling description**"). For each sample we performed the acidity-based sequential solid phase extraction (SSPE) where the sample was first adjusted (i.e., acidified SSW or basified for PPW) to pH 6 and extracted. DOM that was retained on the PPL cartridge during this step was eluted with methanol and termed pH 6 extract. The permeate from the first extraction was collected and acidified to pH 4 and extracted. DOM retained on the PPL cartridge is called pH 4 extract and the permeate is acidified to pH 2 and extracted again. For each extract we performed the derivatization through a deuteromethylation reaction where the carboxylic acids functional groups (COOH-groups highlighted in pink) were deuteroesterified. Each fraction was derivatized and measured separately with reversed-phase (RP) LC-FT-ICR MS without further extraction. The chemical structure in the figure is a representation of a proposed DOM molecule to exemplify in which part of the molecule the derivatization will occur. 8

Figure SI 2 – Schema of all analysis steps performed to the samples. After sampling, DOC concentration was measured via the HTCO method to calculate carbon recoveries. The sample processing further comprises acidity-based SSPE and derivatization (for details see **Fig. SI 1**). LC-FT-ICR MS, ^{1}H NMR, Radiocarbon analysis, and Electrochemistry were performed on the acidity-based SSPE extracts while LC-FT-ICR MS was additionally performed on the derivatized extracts. The LC-FT-ICR MS data processing details is shown in **Fig. SI 3**. 9

Figure SI 3: Data processing workflow outline applied to both extracted and derivatized samples, aiming to refine molecular formula (MF) datasets. The general filtering – applied to both extracted and derivatized samples – involves the subtraction of blank signals, exclusion exclusion of contaminants, and the removal of MF corresponding to specific formulas classes ("CHOS," "CHNOS," and "CHNO") with a *m/z* > 550 (high molecular weight), which are likely wrong assignments. For derivatized samples, an extra filtering (2H filters) was elaborated. Only the MF with 0, 3, 6, 9, 12, 15, 18, or 21 × 2H that meet chemical relationship of three 2H to 2 O are kept. This filtering step ensures that only derivatized molecules adhering to expected isotopic labelling patterns are retained for further analysis. The final filtering steps focus on verifying the robustness and consistency of molecular formula assignments. The workflow ensures to keep only MF found in 2 of 3 replicates, while excluding multiple assignments based on median values derived from existing literature.[3] Additionally, continuity in derivatization series is maintained, an only derivatized MF that correspond to non-derivatized counterparts from the final list of MF from extracts are included. 10

3. Method assessment: acidity-based SSPE 11

3.1. Details on method assessment of acidity-based SSPE 11

3.2. Results on method assessment of acidity-based SSPE 12

Table SI 1 – Acidity-based SSPE carbon yield data containing the experimental pH (Exp. pH), TOC in mg L^{-1} in methanol fractions, yield of the pooled extracts (%), and carbon : PPL mass ratio (C:PPL (%)) for peat pore water sample (PPW) and surface sea water sample (SSW). The total yield of the 3 subsequently extractions combined (Total Yield (%)), and the yield of sample extraction only at pH 2 (direct pH 2, 50 mg PPL[4]) are provided in the bottom row. Since SSW was extracted on four (for pH 6 and pH 4) and three (for pH 2) PPL cartridge in parallel, yields were calculated after pooling the methanol extracts for each pH. The individual yields can be observed in **table SI 2** as repeatability of the extraction. 14

Table SI 2– Repeatability and robustness of acidity-based SSPE TOC data. Experimental pH (Exp. pH), TOC in mg L^{-1} in methanol fractions, yield of the extracts (%) for SSW and Holtemme spring water sample (HSW). The yield of the combined parallel extractions (SSW, HSW) and average of different extraction pH experiments (HSW) is provides as mean and standard deviation (sd). 15

Figure SI 4 – Left: Robustness of acidity-based SSPE was tested by performing hierarchical cluster analysis of different RTs of the pHs 5.8, 6.1 × 3, and 6.6 of the HSW. **Right**: Intermediate precision of SSPE assessed by density plots of the coefficient of variance (CV, %) of the absolute peak intensity of the molecular formulas found more than two times in sample triplicate (pH 2, green) and quadruplicates (pH 4, yellow, pH 6, blue); the dashed lines represent the median and the solid lines the 25th and 75th quantiles. All processed RTs are combined in this analysis. The SSPE-LC-FT-ICR MS showed high intermediate precision, with the highest mean of CV 20.4% for pH 4. After performing the extraction at in slightly different pHs from 5.8 to 6.6 of a terrestrial DOM source (Holtemme spring water sample, HSW), LC-FT-ICR MS and hierarchical cluster analysis we observed high similarity between the same RT of the slightly different pHs. Therefore, the acidity-based fractionation showed to be robust to small pH changes. 16

Figure SI 5 – DOM elution profiles. Total ion chromatograms (TIC) of LC-FT-ICR MS from SSPE extracts of **A.** PPW and **B.** SSW at pH6 (green), pH4 (yellow), pH 2 (blue). Directly extracted samples at pH 2 are overlaid as red trace. [9] Here, polarity is considered directly related to retention since highly polar DOM compounds elute in early retention times (RTs) while low polar DOM compounds elute in later RTs [10,11] 16

4. Method assessment: COOH derivatization reaction 17

Table SI 3 – TOC (mg L^{-1}) and carbon yield (%) after derivatization with deuterated methanol. Each pH extract of peat pore water sample (PPW) and surface sea water sample (SSW) was subjected to the derivatization reaction separately after the extracts were pooled. 19

Figure SI 6 – Extracted ion chromatogram of four CRAM synthesized model compounds.[13] The first row represents the parent compound in the standard methanolic solution. The second row the parent m/z extracted from the derivatized sample. Any of the parent compound was observed in the derivatized samples indicating the they were completely consumed. Since the reaction yield is not 100%, we observe a series of labelled compounds i.e. with one, two, three (for compounds B and D) and one, two, three and four (for compounds A and C). 22

Table SI 4 – Elemental compositions of deprotonated molecules [M-H]- of CRAM model compounds and LC-MS results (error in ppm, intensity of the averaged mass peak in the given retention time (RT) range) 22

Figure SI 7 - Distribution of molecular formulae (MF), with different numbers of COOH-groups per MF obtained from derivatization experiment (x-axis) against number of oxygens. The colour gradient represents the percentage of MF increase based on presence/absence from the 1st to the 2nd consecutive derivatization. Given that we observed only a small increase in yield after additional

derivatizations, we conclude that the first derivatization step successfully accounted for the upper limit of COOH-groups in the majority of MFs. The derivatization method was previously accessed for terrestrial and soil DOM and also has shown to account for the upper limit of most terrestrially derived DOM[14,17] 23

Figure SI 8 - Distribution of molecular formulas (MF) with different numbers of COOH-groups per MF obtained from derivatization experiment (x-axis) separated by 100 Da wide mass bins. The colour gradient represents percentage of MF found in each bin. 24

Figure SI 9 - Isomeric and labelled series behaviour of the DOM molecular formula **$C_{18}H_{22}O_8$** observed in PPW at pH 6. Each bar represents the absolute intensity of a derivatized counterpart of the parent MF **$C_{18}H_{22}O_8$**, with varying numbers of CD_3-groups, corresponding to the derivatization of up to four COOH-groups. At least seven isomers were detected, each with a distinct retention time and a series of labelled derivatives (ranging from 1×CD_3 to 4×CD_3). It is important to note that the differentiation between series members and isomers cannot be determined solely based on this data; this distinction is shown here for illustrative purposes. 25

Figure SI 10: Derivatized DOM elution profiles. Total Ion Chromatogram (TICs) for all derivatized samples measured via LC-FT-ICR MS. A PPW and B SSW 26

5. Electrochemistry 27

5.1. Details on Electrochemical determination of electron- accepting and -donating capacities 27

5.2. Results of Electrochemical determination of EAC and EDC 28

Table SI 5 – Electrochemically determined values for EAC, EDC and calculated oxidation index (OI) for samples (SSW and PPW) extracts at pH 6, 4, and 2. 28

6. Radiocarbon 29

6.1. Details on Radiocarbon analysis 29

6.2. Results on Radio carbon analysis 29

Table SI 6 – Radiocarbon data for PPW and SSW 29

7. Results and discussion 30

Figure SI 11 – Van Krevelen diagram of MFs found in pH 2 (blue), pH 4 (yellow) and pH 6 (green) for PPW (left) and SSW (right). The marginal histograms represent the count of MFs. The weight average (WA) are depicted with diamonds at the respective SSPE (pH) color. All the segments of each extract were summed. 30

Figure SI 13 – Double bond equivalent to carbon ratio (DBE/C_{exp}) distribution of SSW (left, darker colors) and PPW (right, light colors) for sequential solid-phase extraction (SSPE) at pH 2 (blue), pH 4 (yellow) and pH 6 (green). The dotted lines indicate the modes i.e. most repeated values of dispersity indices. 31

Figure SI 14 – Double bond equivalent to carbon ratio (DBE/C_{exp}) distribution of number of COOH-groups per MF for PPW (A) and SSW (B) colored by COOH-group 32

Figure SI 15 - Distribution of DBE/C across different mass windows for each source and group. The upper panel represents PPW, while the lower panel represents SSW. The mean values are indicated by black dots. 33

Figure SI 16 - Distribution of oxygens atoms not bounded as carboxylic acid groups (non-COOH-O) against the number of COOH obtained experimentally by derivatization. The heat map is colored by

the number of carbon atoms not bound in COOH-groups (non-COOH-C). In the pH 6 fraction of SSW, an increasing number of non-COOH-C per MF was observed, which was supported by the increase in alkyl functionalities seen in the 1H NMR (Figure 1 D). Together with the overall low number of COOH-groups at pH 6, this suggests that these compounds were predominantly fatty acids and wax-related carotenoids—expected degradation products of aquatic primary producers in surface seawater.[42,48] 33

Figure SI 17 – Density of dispersity indexes for each COOH-group. PPW can be found on the left and SSW on the right. Peak at dispersity index 3.02 (pH 4 and 6) indicates high frequency of MFs with COOH-groups for PPW. 34

Figure SI 18 – Dispersity indexes density plotted against the **a.** number of O and **b**. DBE-O. The upper row represents the SSW while the bottom PPW. The left column is pH 2, the second pH 4 and the third pH 6. 35

Figure SI 19 – Structural isomeric information on negative (IDEGNEG and positive (IDEGPOS) degradation indices. Extracted ion chromatograms (EICs) are shown for PPW (top) and SSW (bottom) at pH levels of 6 (green), 4 (yellow), and 2 (blue). The bar plots represent the number of carboxylic acid groups (COOH-groups) found *per* molecular formula (MF), as determined by derivatization. "n" indicates the number of isomers found at each pH, and "disp." refers to their dispersity index. Number of MFs “-1” means there is one more isomer detected in the labeled samples compared to the total number of MF in the non-labeled extract. The ten I_{DEG} MF (5 NEGIDEG and 5 POSIDEG) are consistently detected across DOM samples. I_{DEG} is an empirical proxy for the relative age or degradation state of an individual sample within a given set of samples. Concerning their molecular composition, NEG_{IDEG} MF have a lower hydrogen-to-carbon (H/C) ratio compared to POS_{IDEG} MF (but no difference in O/C ratios) and are part of the MF that were suggested to represent CRAM.[23,24] 39

1. Method details

1.1. Sample sites and sampling description

Peat pore water (PPW) was collected in February 2022, surface sea water (SSW) in June 2022. The peatland is an ombrotrophic raised bog, thus, the soil vegetation obtains most of the nutrition from rain water instead of springs or rivers.[1] The organic matter accumulated in anoxic conditions and formed by the degradation of the plant litter justifies the low pH (4.3). The SSW was collected on the mouth of the river Elbe. Thus, the organic matter is formed from autochthonous and allochthonous as primary production, and biodegradation.[2]

The samples were collected in pre-combusted (400 ◦C, 4 h) glass bottles and filtrated through GF/F filters (0.7 µm, Whatman). The PPW was stored at room temperature to avoid DOM precipitation for one day before extraction, and SSW was readily extracted. Dissolved organic carbon (DOC) was measured in both samples with the HTCO method (DIMATOC 2100, Dimatec Analysentechnik, Essen, Germany) following DIN EN 12260 (TOC). The TOC value for PPW is 69.01 mL L^{-1} while SSW is 1.50 mg L^{-1}.

1.2. LC-FT-ICR MS and data treatment

Chromatograms were averaged into 8 (between 13 and 20 min for SSW 2, 14 and 21 min for SSW 4, 12 and 19 for PPW 2, 13 and 20 min for PPW 4), or 10 (between 15 and 24 min for SSW 6, 14 and 23 min for PPW 6) one-minute-long segments (31 scans per segment) and treated as one spectrum. The choice of these settings was based on the distinct DOM elution profiles (see **Figure SI 5**). Peak picking signal-to-noise threshold was set to 4 for all measurements, and each spectrum was internally calibrated with an in-house list of commonly detected DOM mass peaks[3]. For the derivatized fractions, the measurements were internally calibrated with and adjusted mass list, reflecting the high number of deuterium atoms in molecules. Calibration root-mean-squared mass error was less than 0.2 ppm. These analyses were performed in the software DataAnalysis 5.0 (Bruker Daltonics, Billerica, U.S.A.).

After the re-calibration, MF of the SSPE samples were assigned with the Lambda-Miner[4] according to the following rules: $C_{1–60}$, $H_{1–122}$, $O_{0–40}$, $N_{0–4}$, $S_{0–2}$; 0.3 < H/C < 3, 0 < O/C < 1.2, 0 < N/C < 1.5, 0 < DBE (double bond equivalent) < 25.[5] For the derivatized samples,

assignments were done with additional $D_{0\text{-}21}$. RT_{wa} is the weighted average retention time (RT) of all DOM components, reflecting their overall chromatographic distribution.

To limit false assignments due to the large number of D and to increase the confidence of the results, derivatized samples were injected in triplicate and only the MF shared among at least two of the triplicates were considered. In addition, all formulas found in blanks were removed from the derivatized and underivatized fraction measurements. Methyl esters of derivatized samples ~(CD3)x (x = 3, 6, 9, 12, 15, 18, 21) MF were only considered if they were in a continuous series including the monoisotopologue (x = 0). For the derivatized samples, an extra filtration step based on mass error distributions was applied to limit the number of multiple assignments.[6]

1.3. ^{1}H NMR

1.1 mg of C of each SSPE extract was dried in a vacuum centrifugal concentrator (ThermoFisher scientific, Massachusetts, U.S.A.) at 2 mbar for 12 h and freeze dryer (Martin Christ, Osterode am Harz, German) at 0.7 mbar for 48 h. The extracts were redissolved in 200 µL of methanol-D_4, centrifuged at 4000 g in pre-combusted glass micro-inserts, transferred to the NMR tubes with minimal air headspace and sealed. NMR experiments were performed in 1.7 mm microtubes at 292 K with a 600 MHz NMR spectrometer (AVANCE II, Bruker, Bremen, German) equipped with a CPTCI microcryoprobe. Chemical shift referencing was performed against 0.03% tetramethylsilane. Standard pulse programs were used. Proton spectra were acquired with ns = 128, aq = 3 s and d1 = 7 s.

2. Sample preparation and analysis workflow

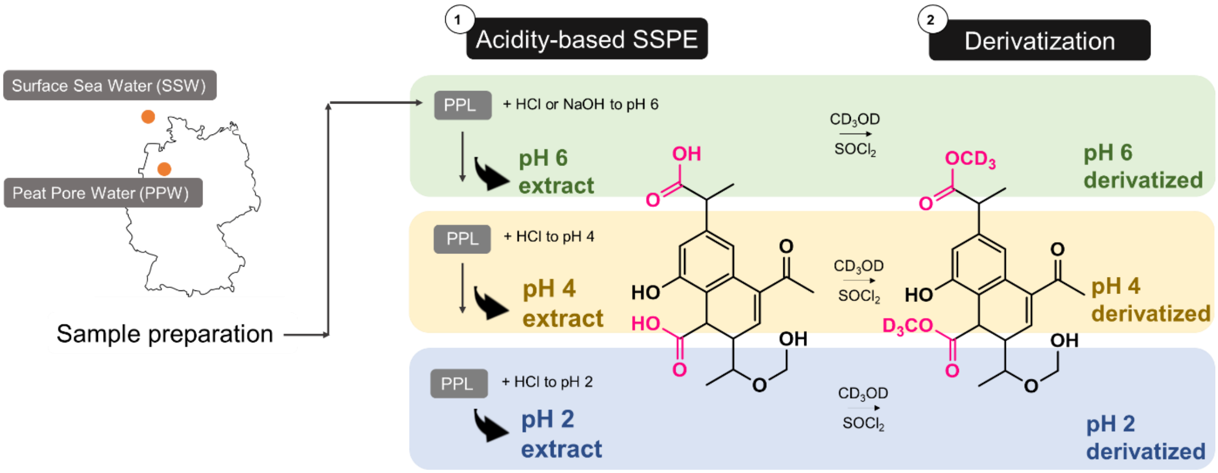

Figure SI 1 – Sample location and sample preparation procedure (acidity-based SSPE and derivatization). Peat pore water (PPW) and surface sea water (SSW) samples were obtained in Germany (as described in “**SI Section 1: Sample sites and sampling description**”). For each sample we performed the acidity-based sequential solid phase extraction (SSPE) where the sample was first adjusted (i.e., acidified SSW or basified for PPW) to pH 6 and extracted. DOM that was retained on the PPL cartridge during this step was eluted with methanol and termed pH 6 extract. The permeate from the first extraction was collected and acidified to pH 4 and extracted. DOM retained on the PPL cartridge is called pH 4 extract and the permeate is acidified to pH 2 and extracted again. For each extract we performed the derivatization through a deuteromethylation reaction where the carboxylic acids functional groups (COOH-groups highlighted in pink) were deuteroesterified. Each fraction was derivatized and measured separately with reversed-phase (RP) LC-FT-ICR MS without further extraction. The chemical structure in the figure is a representation of a proposed DOM molecule to exemplify in which part of the molecule the derivatization will occur.

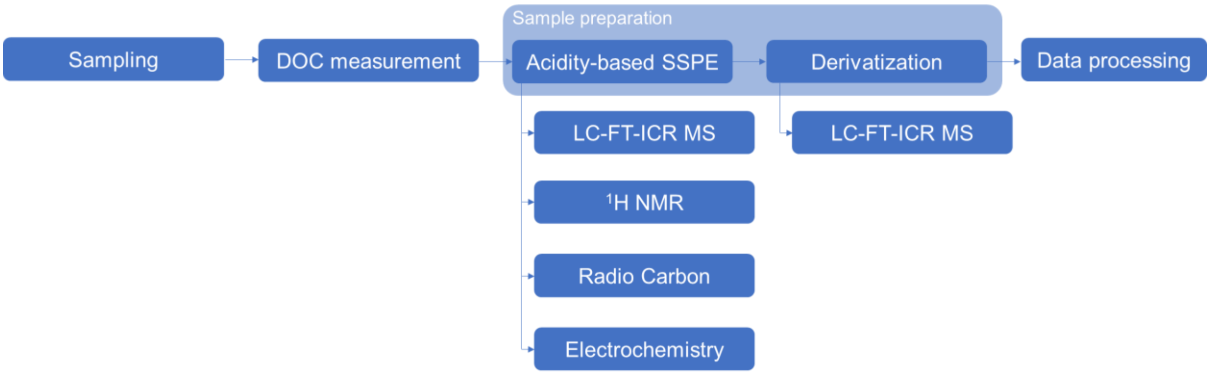

Figure SI 2 – Schema of all analysis steps performed to the samples. After sampling, DOC concentration was measured via the HTCO method to calculate carbon recoveries. The sample processing further comprises acidity-based SSPE and derivatization (for details see **Fig. SI 1**). LC-FT-ICR MS, ^{1}H NMR, Radiocarbon analysis, and Electrochemistry were performed on the acidity-based SSPE extracts while LC-FT-ICR MS was additionally performed on the derivatized extracts. The LC-FT-ICR MS data processing details is shown in **Fig. SI 3**.

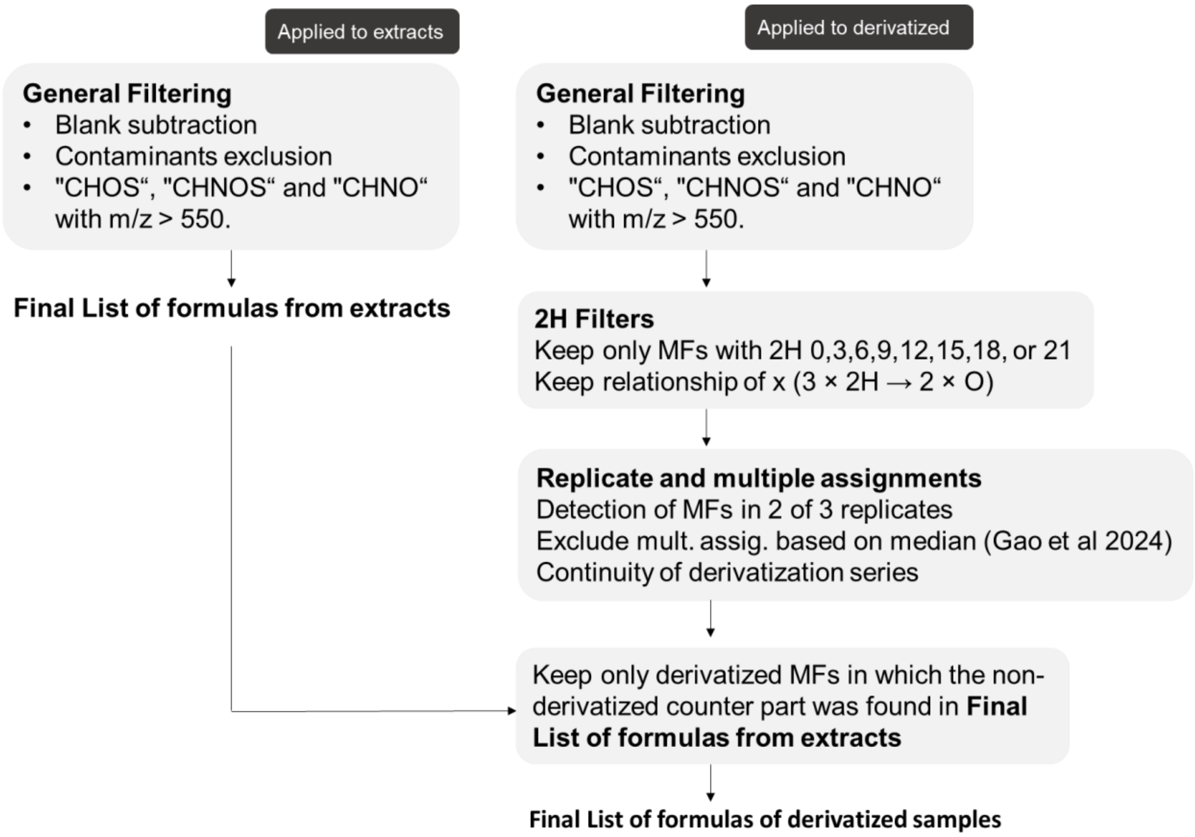

Figure SI 3: Data processing workflow outline applied to both extracted and derivatized samples, aiming to refine molecular formula (MF) datasets. The general filtering – applied to both extracted and derivatized samples – involves the subtraction of blank signals, exclusion exclusion of contaminants, and the removal of MF corresponding to specific formulas classes ("CHOS," "CHNOS," and "CHNO") with a *m/z* > 550 (high molecular weight), which are likely wrong assignments. For derivatized samples, an extra filtering (2H filters) was elaborated. Only the MF with 0, 3, 6, 9, 12, 15, 18, or 21 × 2H that meet chemical relationship of three 2H to 2 O are kept. This filtering step ensures that only derivatized molecules adhering to expected isotopic labelling patterns are retained for further analysis. The final filtering steps focus on verifying the robustness and consistency of molecular formula assignments. The workflow ensures to keep only MF found in 2 of 3 replicates, while excluding multiple assignments based on median values derived from existing literature.[6] Additionally, continuity in derivatization series is maintained, an only derivatized MF that correspond to non-derivatized counterparts from the final list of MF from extracts are included.

3. Method assessment: acidity-based SSPE

An UHPLC system (UltiMate™ 3000, ThermoFisher scientific, Massachusetts, U.S.A.) hyphenated to an FT-ICR-MS with a dynamically harmonized analyzer cell (solariX XR, Bruker Daltonics, Billerica, U.S.A.) and a 12 T refrigerated actively shielded superconducting magnet (Bruker Biospin, Wissembourg, France) were used for all measurements. All samples were normalized before injection to a carbon concentration of 10 mg L^{-1}. The LC method with a counter gradient was adapted from.[7] Briefly, a C_{18} column (ACQUITY HSS T3, 1.8 µm, 100 Å, 150 × 3 mm, Waters, Milford, U.S.A.) equipped with a guard column (ACQUITY UPLC HSS T3 VanGuard, 100 Å, 1.8 µm, 2.1 mm × 5 mm, Waters, Milford, U.S.A.) was used for DOM separation. The solvents of both pumps were MQW (A) and methanol (B). On pump A, the solvents were spiked with 0.1% of formic acid, while on pump B the solvents were spiked with the post-column infused internal standard (PCI-IS) Naproxen-D_3 (50 or 5 ng nL^{-1} depending on the IAT). Suwannee River Fulvic Acid standard (SRFA at 10 mg L^{-1} C, 2S101H) spiked with model compounds was used as quality control.

For full scan acquisition, data were obtained in negative mode with an electrospray ionization source (Apollo II, Bruker Daltonics, Billerica, U.S.A.). The parameters for this method were set as the following: a) capillary voltage equal to 4.3 kV; b) nebulizer gas pressure equal to 1.0 bar; c) dry gas temperature equal to 250 ◦C, and d) dry gas flow rate equal to 8.0 L/min. Since each SSPE and derivatized fraction has a different composition – which drastically changes the ionization efficient, the ion accumulation time (IAT) was adapted for each measurement so that the absolute intensity of the most intense DOM peak of each chromatogram was set $(0.50 \pm 0.30) \times 10^6$. The mass range for acquisition was *m/z* 147-1000. LC-FT-ICR MS total ion chromatograms (TIC) of fractions and derivatized samples can be found in **Figure SI 5** and **Figure SI 9.**

3.1. Details on method assessment of acidity-based SSPE

On a bulk level, to understand how much carbon was lost or gained with the SSPE extraction,a the samples were also extracted according to the standard extraction method [8]. Briefly, PPW and SSW were acidified to pH 2 and loaded into 50 mg PPL cartridges previously conditioned with 2 × 1 mL of methanol and 2 × 1 mL of MQW. The cartridges were washed

with 2 × 1 mL of MQW and dried with N_2 flow before the elution with 3 × 1 mL of methanol. TOC of the fractions were also measured as mentioned above (**Table SI 1**).

To evaluate the intermediate precision of the method, 60 L of SSW were extracted as sample quadruplicate at pH 6, and 4 and as triplicate at pH 2 (**Table SI 2**). Aliquots of fractions and permeates were analyzed for TOC as abovementioned and injected at the LC-FT-ICR MS at 10 mg L^{-1} C, separately. Before the COOH labelling experiment, replicates were pooled together. The robustness of the SSPE was evaluated by extraction of Holtemme river sample (HRW; details on the source and sampling in [9]) at pH 6 – the most susceptive to pH variations. In brief, HRW's pH was adjusted to pH 5.8, 6.1 × 3, and 6.6 with NaOH 2 M, extracted with 50 mg PPL cartridges and eluted as previous described. The eluates were measured for DOC and injected at the LC-FT-ICR MS at 10 mg L^{-1} C. For both experiments the fractions were injected with 200 ms IAT.

For the data processing of the intermediate precision, the chromatograms were averaged into 5 one-minute segments (between 15 and 19 min for pH 2; 16 and 20 min for pH 4 and 18 and 22 min for pH 6). For robustness, the chromatograms were averaged into 5 (between 17 and 21 min) one-minute segments which corresponds to the main DOM elution RTs. The LC-FT-ICR MS results were recalibrated and assigned for MFs as previous mentioned. The final set of MFs was obtained after filtration of MFs found in the blank sample. For the intermediate precision, the variability between the sample replicates were evaluated by the coefficient of variance of the peak intensity of MFs found more than two times in quadruplicates (pH 4 and 6) and triplicates (pH 2). For the robustness, to evaluate the similarity of the pHs, a hierarchical cluster analysis based on the Bray–Curtis dissimilarity [10] was performed with the "vegan" package in R studio (version 2023.09.1+494). For both evaluations, intermediate precision and robustness, the absolute abundances of all MFs of the final set were used to visualize RTs similarities. The analysis was performed with all the MFs absolute intensity of the treated RTs and the complete-linkage clustering approach was chosen given its advantage of separating clusters even in the presence of noise. Slight variations in pH during extraction did not appear to impact the elution profiles or the molecular signature of the DOM (**Table SI 2, Figure SI 4**).

3.2. Results on method assessment of acidity-based SSPE

The carbon recovery of the acidity-based SSPE was compared to the standard pH 2 extraction for both PPW and SSW samples. SSPE yielded significantly higher recoveries, with PPW and SSW achieving 70% compared to 49% and 50% for the standard extraction at pH 2

only, respectively (**Table SI 1**, and **Table SI 2**). This highlights the effectiveness of SSPE in recovering a broader range of DOM compounds in both sample types, achieving better representativeness of the bulk DOM composition. [8] To ensure that no contamination or loss of DOM during the SSPE process biases the recovery assessment, a mass balance was calculated as the sum of carbon (C) in the methanol extracts and permeates divided by the total carbon in the original sample for each pH. (equation 1).

$$SSPE\ recovery\ (\%) = \frac{C\ SSPE6\ (mg) + C\ permeate_6(mg) + C\ SSPE4\ (mg) + C\ permeate_4(mg) + C\ SSPE2\ (mg) + C\ permeate_2(mg)\ x\ 100}{C\ original\ sample\ (mg)} \quad \text{(Equation 1)}$$

For PPW, the mass balance yielded total recovered C of 94 ± 5% at pH 6, 90 ± 5% at pH 4, and 88 ± 5% at pH 2. Similarly, for SSW, the C recoveries were 92 ± 5% at pH 6, 91 ± 5% at pH 4, and 85 ± 5% at pH 2. Decreasing recoveries at lower pH was likely due to water losses during extraction and transferring steps, which accounted for approximately 4 L out of the 60 L processed sample volumes. These results confirm high recovery rates for DOM across all tested pH levels, with no evidence of procedural contamination. The C:PPL ratios were maintained between 0.35% and 0.41%, optimizing the DOM-PPL physisorption mechanism over self-assembly adsorption. [11] These results support the assumption SSPE is not limited by adsorption capacity (controlling C:PPL ratios) but rather by pKa value.

The robustness of the SSPE method was evaluated by testing its performance under slight pH variations during the extraction of a Holtemme river samples (HSW) – a different sample used exclusively for this analysis. Samples were extracted at pH values of 5.8, 6.1, and 6.6 to mimic practical deviations from the target pH of 6. DOC concentration of each extract was measured (**Table SI 2**) and LC-FT-ICR MS performed. Hierarchical clustering analysis using Bray–Curtis dissimilarity revealed minimal differences in molecular composition across the tested pH levels. The results showed that MFs at the same RTs across different samples were more similar than those at different RTs within the same sample. This finding highlights the high similarity and reproducibility of DOM elution profiles even under slightly varying extraction conditions. The SSPE method demonstrated a high tolerance for minor pH deviations, ensuring consistent molecular characterization even when exact pH adjustments were not achieved. These findings access the robustness of the SSPE method for DOM extraction and analysis under realistic laboratory conditions.

Intermediate precision was assessed by extracting 60 L of SSW at pH 2, 4, and 6 in multiple replicates. Due to the low DOC concentration in SSW, large volumes were required, processed in 4 × 5 g PPL cartridges at pH 4 and 6 and in 3 × 5 g cartridges at pH 2. DOC of

each extract was measured (**Table SI 2**) and LC-FT-ICR MS performed. The repeatability of the SSPE method was already evident from low variability in recovery percentages across replicates for SSW. At pH 6, the average recovery based on carbon was 22% ± 3%, while at pH 4 and pH 2, the average yields were 28% ± 4% and 46% ± 3%, respectively. The coefficient of variation (CV%) for the relative intensities of molecular formulas across replicates averaged below 21% for all tested pH conditions (**Figure SI 4**), similar to repeat injections of the same sample in LC-FT-ICR MS.[12] This low variability demonstrates the high reproducibility of the SSPE method, confirming that individual molecular formula intensities remain consistent across replicates. The consistent intermediate precision across pH levels highlights the reliability of the SSPE method for DOM extraction and analysis, making it suitable for rigorous and repeatable molecular characterization of complex environmental samples.

Table SI 1 – Acidity-based SSPE carbon yield data containing the experimental pH (Exp. pH), TOC in mg L^{-1} in methanol fractions, yield of the pooled extracts (%), and carbon : PPL mass ratio (C:PPL (%)) for peat pore water sample (PPW) and surface sea water sample (SSW). The total yield of the 3 subsequently extractions combined (Total Yield (%)), and the yield of sample extraction only at pH 2 (direct pH 2, 50 mg PPL[8]) are provided in the bottom row. Since SSW was extracted on four (for pH 6 and pH 4) and three (for pH 2) PPL cartridge in parallel, yields were calculated after pooling the methanol extracts for each pH. The individual yields can be observed in **table SI 2** as repeatability of the extraction.

	PPW				SSW			
**SSPE Extracts**	**Exp. pH**	**TOC (mg L^{-1})**	**Yield (%)**	**C:PPL (%)**	**Exp. pH**	**TOC (mg L^{-1})**	**Yield (%)**	**C:PPL (%)**
**pH 6**	6.05	103	23	0.37	6.01	82	21	0.45
**pH 4**	3.99	109	27	0.41	4.03	79	27	0.35
**pH 2**	2.05	124	32	0.37	2.02	130	47	0.37
**Total Yield (%)**			70				70	
**Direct pH 2**	2.01	34	49	0.41	2.00	69	50	0.41

Table SI 2– Repeatability and robustness of acidity-based SSPE TOC data. Experimental pH (Exp. pH), TOC in mg L^{-1} in methanol fractions, yield of the extracts (%) for SSW and Holtemme spring water sample (HSW). The yield of the combined parallel extractions (SSW, HSW) and average of different extraction pH experiments (HSW) is provides as mean and standard deviation (sd).

	SSW				HSW			
**SSPE Extracts**	**Exp. pH**	**Replicate**	**TOC (mg L^{-1})**	**Yield (%)**	**SSPE Extracts**	**Exp. pH**	**TOC (mg L^{-1})**	**Yield (%)**
pH 6	6.01	1	95	25	pH 6.6	6.65	53.5	27
		2	85	23	pH 6.1-1	6.08	55	27
		3	70	19	pH 6.1-2	6.08	51.5	26
		4	78	21	pH 6.1-3	6.09	54.5	27
		mean ± sd		22 ± 3	pH 5.8	5.84	57	28
pH 4	4.02	1	95	33	mean ± sd			27 ± 1
		2	75	26				
		3	76	26				
		4	76	26				
		mean ± sd		28 ± 4				
pH 2	2.00	1	121	43				
		2	132	47				
		3	138	49				
		mean ± sd		46 ± 3				

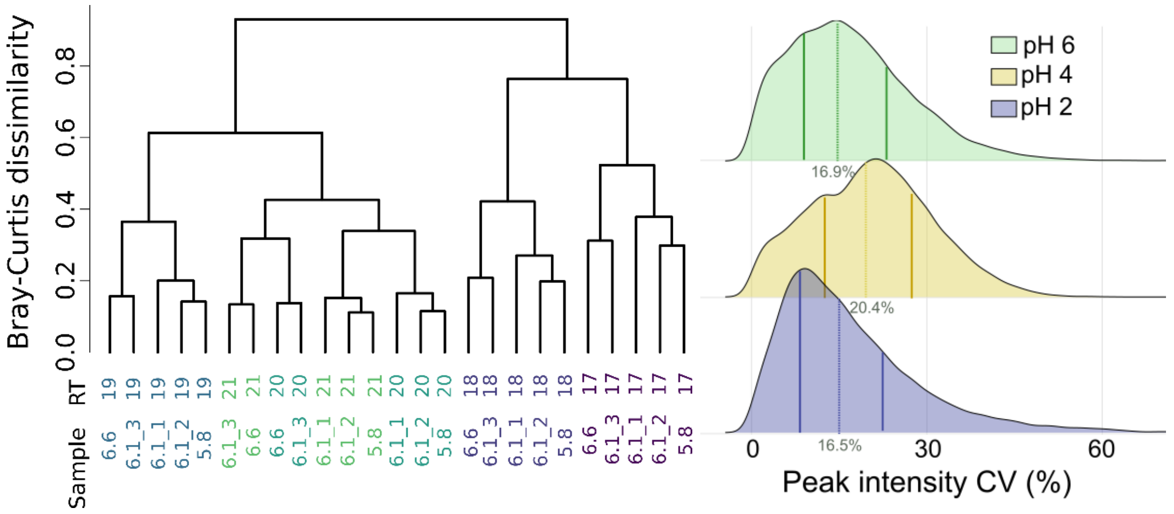

Figure SI 4 – Left: Robustness of acidity-based SSPE was tested by performing hierarchical cluster analysis of different RTs of the pHs 5.8, 6.1 × 3, and 6.6 of the HSW. **Right**: Intermediate precision of SSPE assessed by density plots of the coefficient of variance (CV, %) of the absolute peak intensity of the molecular formulas found more than two times in sample triplicate (pH 2, green) and quadruplicates (pH 4, yellow, pH 6, blue); the dashed lines represent the median and the solid lines the 25th and 75th quantiles. All processed RTs are combined in this analysis. The SSPE-LC-FT-ICR MS showed high intermediate precision, with the highest mean of CV 20.4% for pH 4. After performing the extraction at in slightly different pHs from 5.8 to 6.6 of a terrestrial DOM source (Holtemme spring water sample, HSW), LC-FT-ICR MS and hierarchical cluster analysis we observed high similarity between the same RT of the slightly different pHs. Therefore, the acidity-based fractionation showed to be robust to small pH changes.

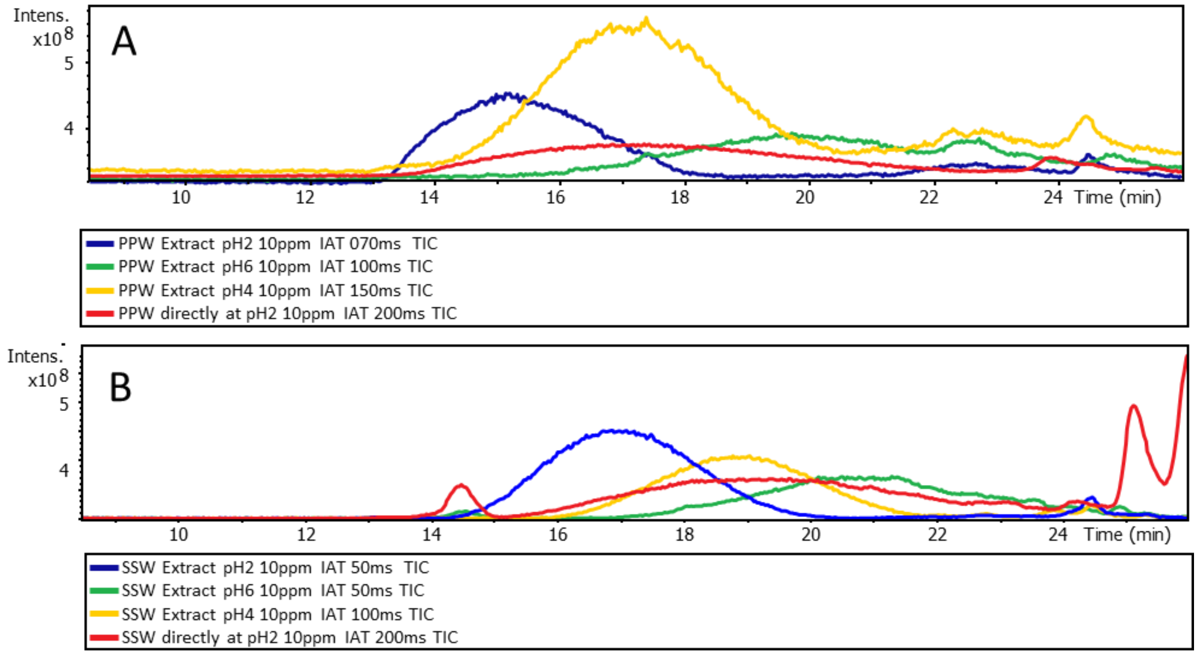

Figure SI 5 – DOM elution profiles. Total ion chromatograms (TIC) of LC-FT-ICR MS from SSPE extracts of **A.** PPW and **B.** SSW at pH6 (green), pH4 (yellow), pH 2 (blue). Directly extracted samples at pH 2 are overlaid as red trace. [13] Here, polarity is considered directly related to retention since highly polar DOM compounds elute in early retention times (RTs) while low polar DOM compounds elute in later RTs [14,15].

4. Method assessment: COOH derivatization reaction

4.1. Details on method assessment COOH derivatization reaction

To understand the completeness of the derivatization reaction, the seawater DOM reference material TRM-0522[16] was consecutively submitted to the derivatization reaction (two times) followed by LC-FT-ICR MS analysis. Only a small increase in yield after the second derivatization was observed, indicating the first derivatization step successfully accounted for the upper limit of COOH-groups in the majority of MFs (**Figure SI 7**). To access if the derivatization approach reaches the upper limit of COOH-groups per compound, four CRAM model compounds[17] were derivatized and analyzed by LC-FT-orbitrap-MS. The maximum number of COOH-groups was obtained for two CRAM model compounds (B and D). The other two CRAM compounds (A and C) probably faced limitation on ionization of the ester, being therefore not detected.

For the CRAM standards analysis, liquid chromatography was carried out on a Thermo Vanquish UPLC equipped with a Phenomenex Kinetex C18 column (2.1 × 150 mm, 1.7 μm) at a temperature of 50 °C and a flow rate of 0.4 mL/min. Mobile phase A consisted of 0.1% formic acid (AnalaR NORMAPUR, VWR) in deionized water (Millipore Milli-Q), while mobile phase B contained 0.1% formic acid in LC-MS grade acetonitrile (Lichrosolv, Supelco, Merck). The gradient began with 5% B, increasing to 95% B from 1 to 10 minutes, followed by a wash at 95% B, a return to 5% B, and a 3.9-minute equilibration phase. The eluate was continuously injected in a mass spectrometry (Thermo Fisher Orbitrap Q Exactive) coupled with electrospray ionization in negative mode, with a heated ESI unit at 150 °C and a −2.5 kV voltage. Sheath and auxiliary gases were set to 15 and 5 units, respectively, the S-Lens to 60, and capillary temperature to 200 °C. The resolution was configured to 70,000, with a maximum injection time of 200 ms to trap approximately 3×10^6 ions.

4.2. Results on method assessment COOH derivatization reaction

After derivatization, the extracts were analyzed for DOC concentration and characterized by LC-FT-ICR MS. Since derivatization adds one carbon for each COOH group by converting them into esters, the carbon yield increased to over 100% for all extracts of both samples compared to their non-derivatized counterparts (**Table SI 3**). This indicates that COOH-groups were successfully derivatized to esters but carbon losses may have happened during the reaction and storage, so the carbon increase cannot be evaluated quantitively.

The derivatization procedure's effectiveness has been previously demonstrated for soil and terrestrial DOM.[18–20] Thus, the focus of this study was to assess its application to marine DOM. All four CRAMs are mixtures of diastereomers, which explains the observation of multiple peaks for the parent standard in the extracted ion chromatograms (EICs) (**Figure SI 6, table SI 4**). In none of the derivatized standard samples was the parent ion observed, indicating complete consumption during derivatization. Only derivatized products were detected.

For compounds B and D (**Figure SI 6)**, the up to the maximum number of COOH-groups were detected as labelled. In contrast, compounds A and C exhibited incomplete labelling, with three labels detected instead of the expected four. This limitation could come from incomplete labelling or undetectability of fully labelled compounds due to the absence of ionizable hydrogens. The derivatization method assumes proportional labelling relative to COOH group availability, but these results suggest a potential inability to detect fully labelled counter-parts of certain molecules. Thus, the number of COOH-groups was estimated based on the number of detected esters e.g. one ester corresponds to one COOH-group. For DOM molecules, ionization may still be supported by other functional groups with labile protons (e.g. phenols and alcohols) that were not present in the model CRAM compounds. This is supported by observed full COOH coverage (i.e., all O atoms are detected as COOH) in marine and terrestrial DOM. [21]

Furthermore, the apparent labelling yields can be influenced by functional group arrangement, steric effects, or ionization efficiency. To test, if labelling yield could be improved, the marine reference material TRM-0522 was sequentially labelled (2 times) and results revealed only a 2.75% increase in esterification yield from the first to the second derivatization (**Figure SI 7**), indicating that the first reaction captured the upper limit of COOH-groups for most MFs in marine DOM. During reaction (which is carried out under mild heating conditions and reflux), CD_3OD may interact with other functional groups like ethers, alcohols, and ketones, resulting in a possible overestimation of COOH-groups. While the derivatization method is robust for identifying trends in COOH-group abundance, further work is necessary to validate the proportionality assumption between labelling and COOH content. Complementary techniques, such as NMR, can provide a more comprehensive characterization of DOM functional groups, especially for high-molecular-weight aromatic compounds that are underrepresented in ESI-MS. [13]

For the SSW and PPW samples, SSW exhibited a higher frequency of MFs containing COOH groups at pH 2 and 4 (**Figure SI 8)**. Up to 11.51% of MFs contained three COOH groups at pH 2, and 10.05% had two COOH groups at pH 4. Similarly, between three and four

COOH-groups per MF were observed in North Sea water under the same derivatization procedure. [19] In contrast, terrestrial DOM (PPW) showed distinct characteristics, with most MFs containing between zero and one COOH group. However, a small pool of MFs in PPW appeared highly reduced, with up to 1.45% of MFs containing seven COOH-groups per molecule, compared to only 0.09% in SSW (**Figure SI 8**). These findings are consistent with previous studies, where terrestrial DOM was found to have higher numbers of COOH-groups per molecule than surface marine DOM when analyzed using the same derivatization method. [21] Further evidence comes from fragmentation experiments (FT-ICR MS/MS), which demonstrated that most oxygens in terrestrial DOM molecules were bound as carboxylic acids, as indicated by consecutive CO2 neutral losses. [22] Proposed structures of fulvic acids from other studies have included molecules with up to seven COOH-groups. [23] These observations highlight the complexity and heterogeneity of DOM and underscore the derivatization method's ability to reveal COOH group distributions across diverse environmental samples.

Table SI 3 – TOC (mg L^{-1}) and carbon yield (%) after derivatization with deuterated methanol. Each pH extract of peat pore water sample (PPW) and surface sea water sample (SSW) was subjected to the derivatization reaction separately after the extracts were pooled.

Derivatized Extracts	PPW		SSW	
	TOC (mg L^{-1})	Yield (%)	TOC (mg L^{-1})	Yield (%)
**pH 6**	615	205	282	103
**pH 4**	975	138	331	121
**pH 2**	1830	124	399	126

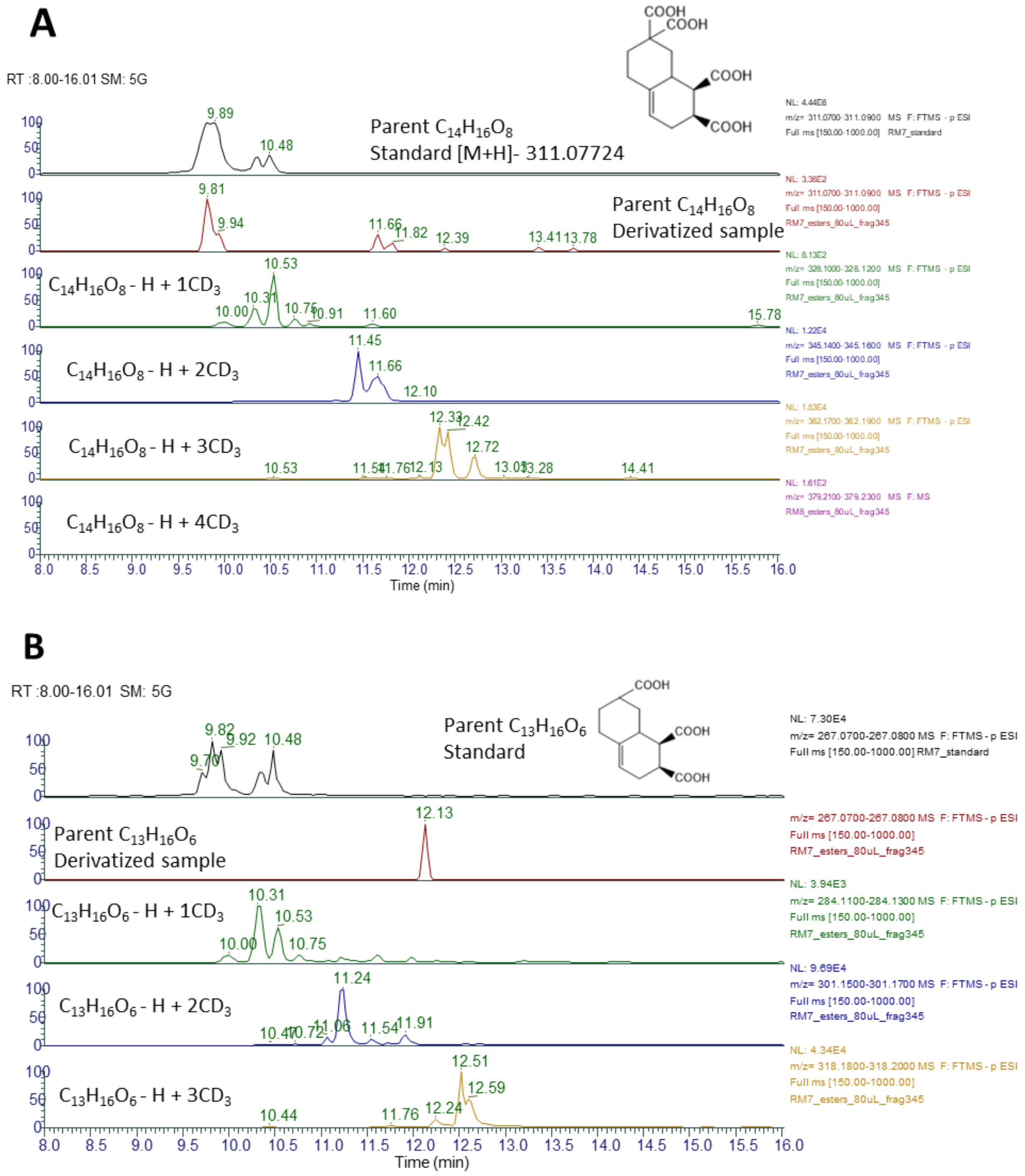
A
RT :8.00-16.01 SM: 5G
Parent C14H16O8
Standard [M+H]- 311.07724
Parent C14H16O8
Derivatized sample
C14H16O8 - H + 1CD3
C14H16O8 - H + 2CD3
C14H16O8 - H + 3CD3
C14H16O8 - H + 4CD3
COOH
COOH
COOH
COOH
9.89
10.48
9.81
9.94
11.66
11.82
12.39
13.41
13.78
10.53
10.31
10.00
10.75
10.91
11.60
15.78
11.45
11.66
12.10
12.33
12.42
12.72
10.53
11.54
11.76
12.13
13.03
13.28
14.41
NL: 4.44E6
m/z= 311.0700-311.0900 MS F: FTMS - p ESI
Full ms [150.00-1000.00] RM7_standard
NL: 3.38E2
m/z= 311.0700-311.0900 MS F: FTMS - p ESI
Full ms [150.00-1000.00]
RM7_esters_80uL_frag345
NL: 8.13E2
m/z= 328.1000-328.1200 MS F: FTMS - p ESI
Full ms [150.00-1000.00]
RM7_esters_80uL_frag345
NL: 1.22E4
m/z= 345.1400-345.1600 MS F: FTMS - p ESI
Full ms [150.00-1000.00]
RM7_esters_80uL_frag345
NL: 1.53E4
m/z= 362.1700-362.1900 MS F: FTMS - p ESI
Full ms [150.00-1000.00]
RM7_esters_80uL_frag345
NL: 1.61E2
m/z= 379.2100-379.2300 MS F: MS
RM8_esters_80uL_frag345
Time (min)
B
RT :8.00-16.01 SM: 5G
Parent C13H16O6
Standard
Parent C13H16O6
Derivatized sample
C13H16O6 - H + 1CD3
C13H16O6 - H + 2CD3
C13H16O6 - H + 3CD3
COOH
COOH
COOH
9.82
9.92
10.48
9.70
12.13
10.31
10.53
10.00
10.75
11.24
10.47
10.72
11.06
11.54
11.91
12.51
12.59
12.24
11.76
10.44
NL: 7.30E4
m/z= 267.0700-267.0800 MS F: FTMS - p ESI
Full ms [150.00-1000.00] RM7_standard
m/z= 267.0700-267.0800 MS F: FTMS - p ESI
Full ms [150.00-1000.00]
RM7_esters_80uL_frag345
NL: 3.94E3
m/z= 284.1100-284.1300 MS F: FTMS - p ESI
Full ms [150.00-1000.00]
RM7_esters_80uL_frag345
NL: 9.69E4
m/z= 301.1500-301.1700 MS F: FTMS - p ESI
Full ms [150.00-1000.00]
RM7_esters_80uL_frag345
NL: 4.34E4
m/z= 318.1800-318.2000 MS F: FTMS - p ESI
Full ms [150.00-1000.00]
RM7_esters_80uL_frag345
Time (min)

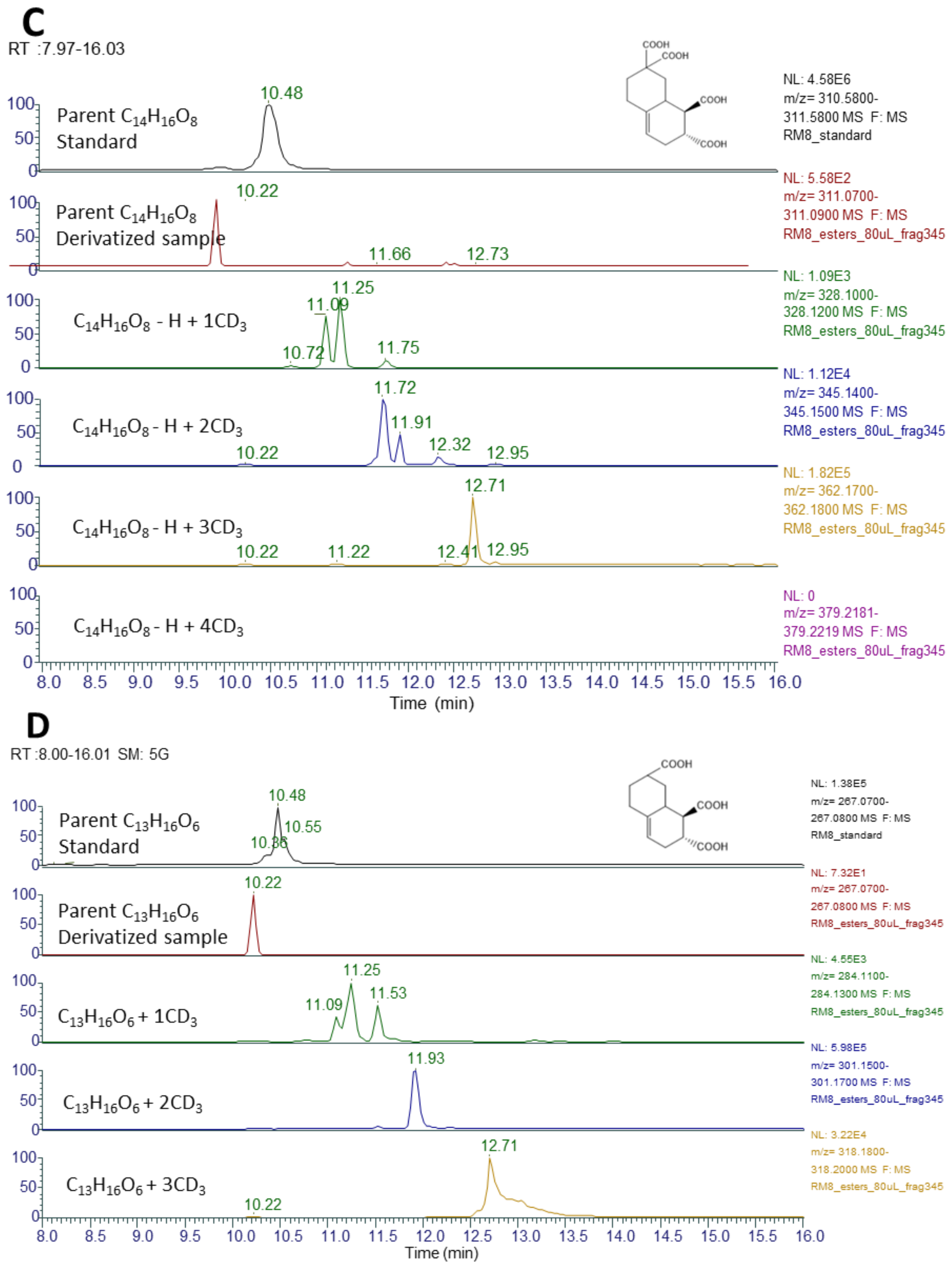

C
RT :7.97-16.03
10.48
Parent C14H16O8
Standard
NL: 4.58E6
m/z= 310.5800-
311.5800 MS F: MS
RM8_standard
10.22
Parent C14H16O8
Derivatized sample
11.66
12.73
NL: 5.58E2
m/z= 311.0700-
311.0900 MS F: MS
RM8_esters_80uL_frag345
11.25
11.09
10.72
11.75
C14H16O8 - H + 1CD3
NL: 1.09E3
m/z= 328.1000-
328.1200 MS F: MS
RM8_esters_80uL_frag345
11.72
11.91
12.32
12.95
10.22
C14H16O8 - H + 2CD3
NL: 1.12E4
m/z= 345.1400-
345.1500 MS F: MS
RM8_esters_80uL_frag345
12.71
10.22
11.22
12.41
12.95
C14H16O8 - H + 3CD3
NL: 1.82E5
m/z= 362.1700-
362.1800 MS F: MS
RM8_esters_80uL_frag345
C14H16O8 - H + 4CD3
NL: 0
m/z= 379.2181-
379.2219 MS F: MS
RM8_esters_80uL_frag345
Time (min)
D
RT :8.00-16.01 SM: 5G
10.48
10.55
10.36
Parent C13H16O6
Standard
NL: 1.38E5
m/z= 267.0700-
267.0800 MS F: MS
RM8_standard
10.22
Parent C13H16O6
Derivatized sample
NL: 7.32E1
m/z= 267.0700-
267.0800 MS F: MS
RM8_esters_80uL_frag345
11.25
11.09
11.53
C13H16O6 + 1CD3
NL: 4.55E3
m/z= 284.1100-
284.1300 MS F: MS
RM8_esters_80uL_frag345
11.93
C13H16O6 + 2CD3
NL: 5.98E5
m/z= 301.1500-
301.1700 MS F: MS
RM8_esters_80uL_frag345
12.71
10.22
C13H16O6 + 3CD3
NL: 3.22E4
m/z= 318.1800-
318.2000 MS F: MS
RM8_esters_80uL_frag345
Time (min)
COOH

Figure SI 6 – Extracted ion chromatogram of four CRAM synthesized model compounds.[17] The first row represents the parent compound in the standard methanolic solution. The second row the parent m/z extracted from the derivatized sample. Any of the parent compound was observed in the derivatized samples indicating the they were completely consumed. Since the reaction yield is not 100%, we observe a series of labelled compounds i.e. with one, two, three (for compounds B and D) and one, two, three and four (for compounds A and C).

Table SI 4 – Elemental compositions of deprotonated molecules [M-H]- of CRAM model compounds and LC-MS results (error in ppm, intensity of the averaged mass peak in the given retention time (RT) range).

	**A** 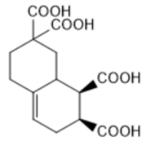				**C** 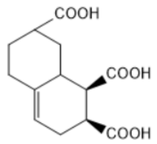			
**Compound**	**[M-H]-**	**Err. (ppm)**	**RT (min)**	**Int.**	**[M-H]-**	**Err. (ppm)**	**RT (min)**	**Int.**
Parent $C_{14}H_{16}O_8$ Standard	311.0771	0.45	9.68-9.98	4.25E+06	267.0874	0.04	9.7-9.99	3.17E+06
Parent $C_{14}H_{16}O_8$ Derivatized sample	311.0751	6.88	9.71-10.00	1.12E+02	-	-	-	-
$C_{14}H_{16}O_8$ - H + 1CD_3	328.1089	8.59	10.49-10.59	5.00E+02	284.1196	8.06	10.26-10.39	2.13E+03
$C_{14}H_{16}O_8$ - H + 2CD_3	345.143	9.27	11.41-11.49	9.17E+03	301.1532	10.53	11.18-11.31	6.57E+04
$C_{14}H_{16}O_8$ - H + 3CD_3	362.1772	9.61	12.29-12.45	1.08E+04	318.1875	10.53	12.5-12.64	2.93E+04
$C_{14}H_{16}O_8$ - H + 4CD_3	-	-	-	-	-	-	-	-
	**B** 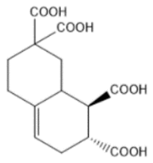				**D** 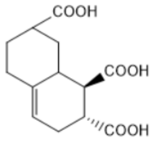			
Parent $C_{14}H_{16}O_8$ Standard	311.0772	900098.12	10.41-10.58	3.21e6	267.0875	-0.34	10.36-10.61	4.98E+06
Parent $C_{14}H_{16}O_8$ Derivatized sample	-	-	-	-	-	-	-	-
$C_{14}H_{16}O_8$ - H + 1CD_3	-	-	-	-	284.1194	8.76	11.17-11.30	3.50E+03
$C_{14}H_{16}O_8$ - H + 2CD_3	345.1433	8.40	11.6-11.8	6.40E+03	301.1531	10.86	11.88-11.98	3.86E+04
$C_{14}H_{16}O_8$ - H + 3CD_3	362.1767	10.99	12.66-12.75	1.31E+05	318.1875	10.53	12.7-12.85	2.30E+04

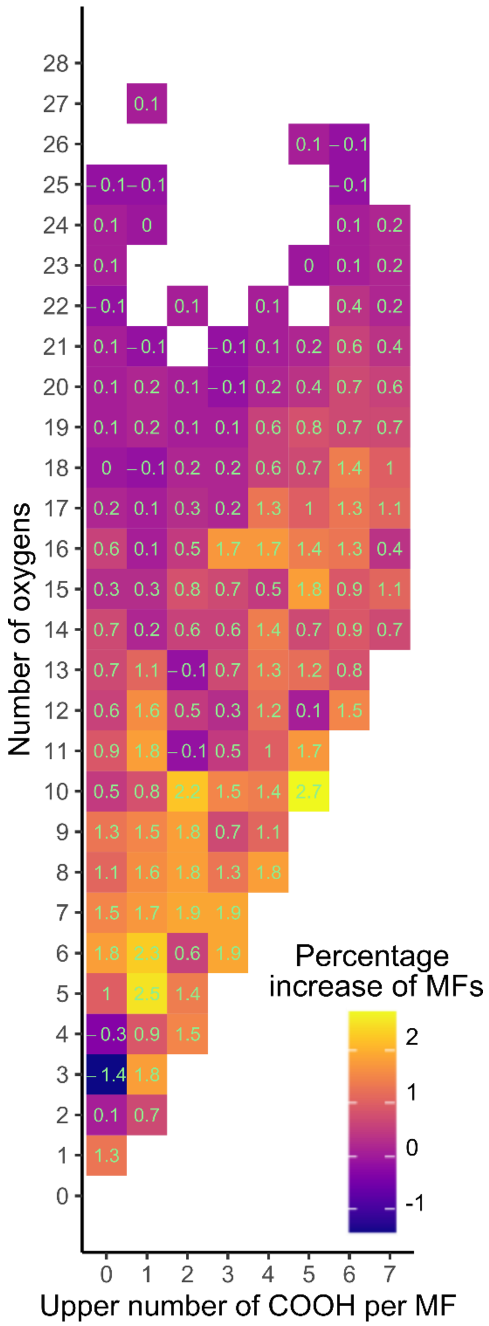

Figure SI 7 - Distribution of molecular formulae (MF), with different numbers of COOH-groups per MF obtained from derivatization experiment (x-axis) against number of oxygens. The colour gradient represents the percentage of MF increase based on presence/absence from the 1st to the 2nd consecutive derivatization. Given that we observed only a small increase in yield after additional derivatizations, we conclude that the first derivatization step successfully accounted for the upper limit of COOH-groups in the majority of MFs. The derivatization method was previously accessed for terrestrial and soil DOM and also has shown to account for the upper limit of most terrestrially derived DOM[18,21]

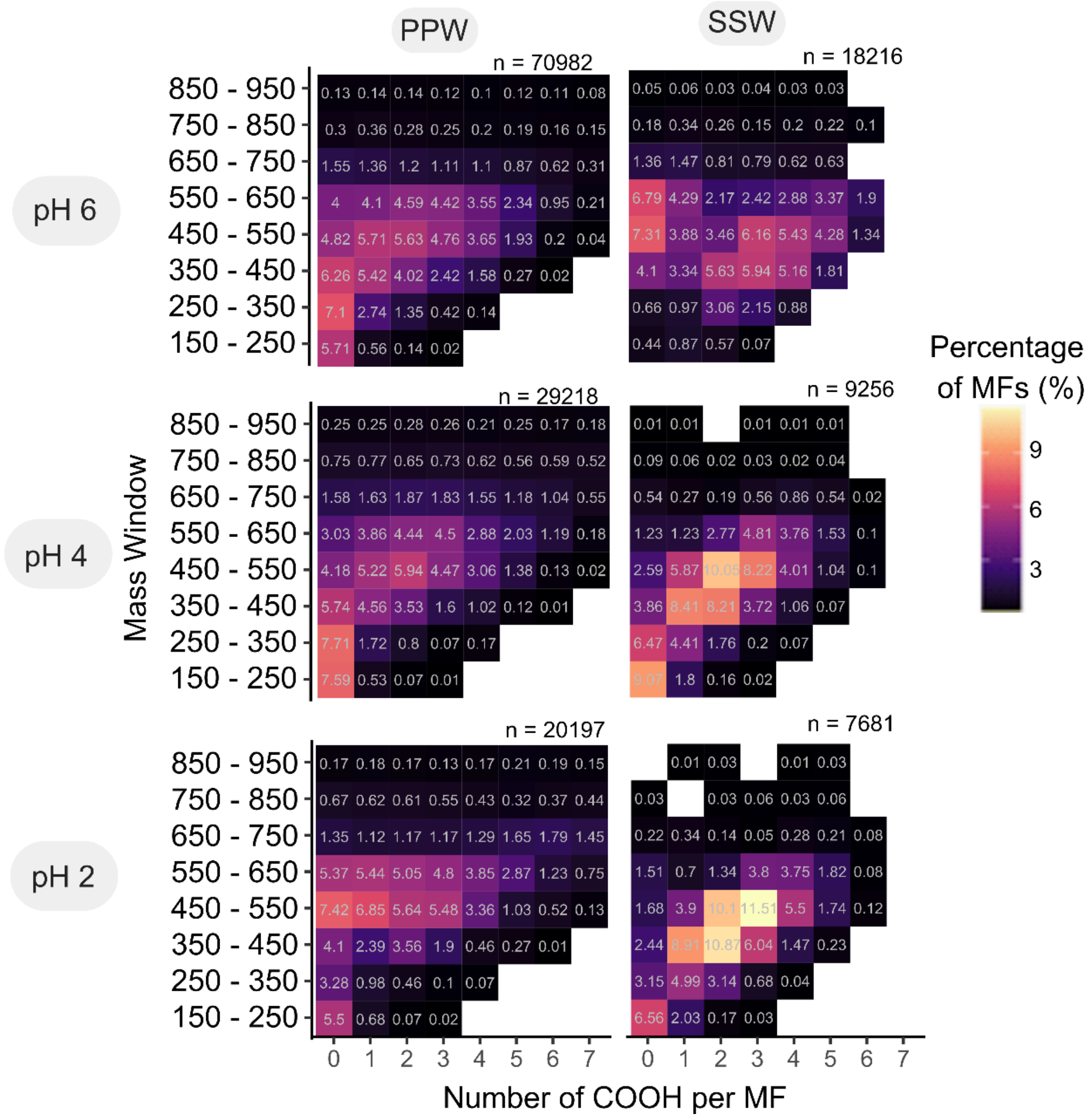

Figure SI 8 - Distribution of molecular formulas (MF) with different numbers of COOH-groups per MF obtained from derivatization experiment (x-axis) separated by 100 Da wide mass bins. The colour gradient represents percentage of MF found in each bin.

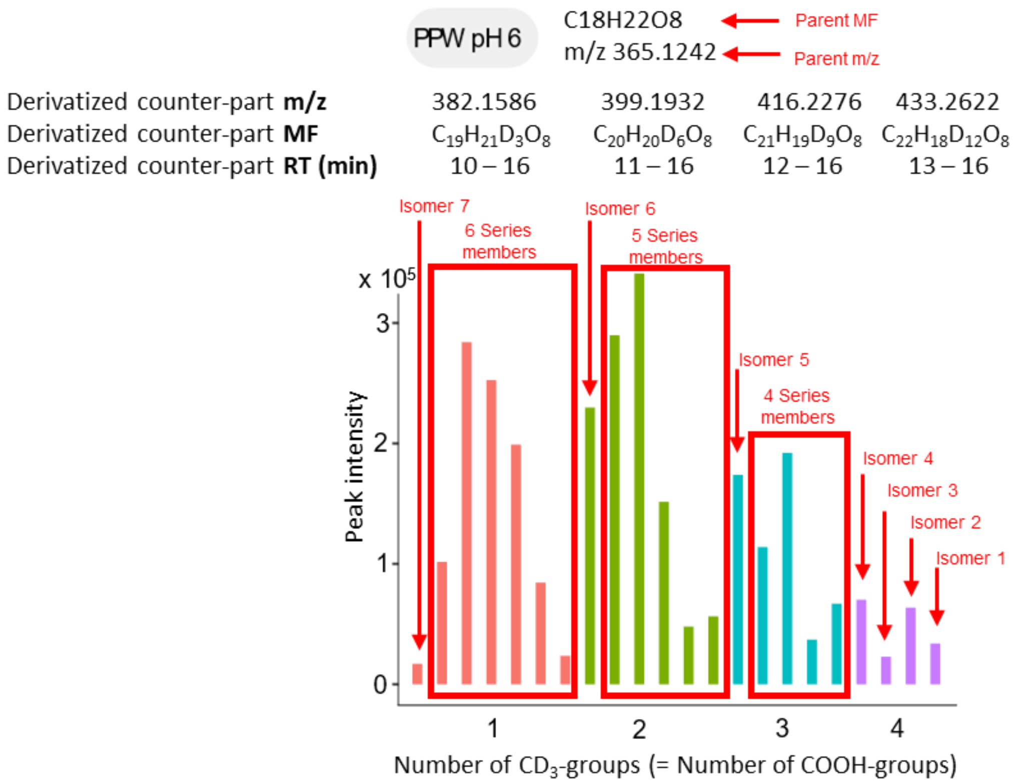

Figure SI 9 - Isomeric and labelled series behaviour of the DOM molecular formula $\mathbf{C_{18}H_{22}O_8}$ observed in PPW at pH 6. Each bar represents the absolute intensity of a derivatized counterpart of the parent MF $\mathbf{C_{18}H_{22}O_8}$, with varying numbers of CD_3-groups, corresponding to the derivatization of up to four COOH-groups. At least seven isomers were detected, each with a distinct retention time and a series of labelled derivatives (ranging from $1{\times}CD_3$ to $4{\times}CD_3$). It is important to note that the differentiation between series members and isomers cannot be determined solely based on this data; this distinction is shown here for illustrative purposes.

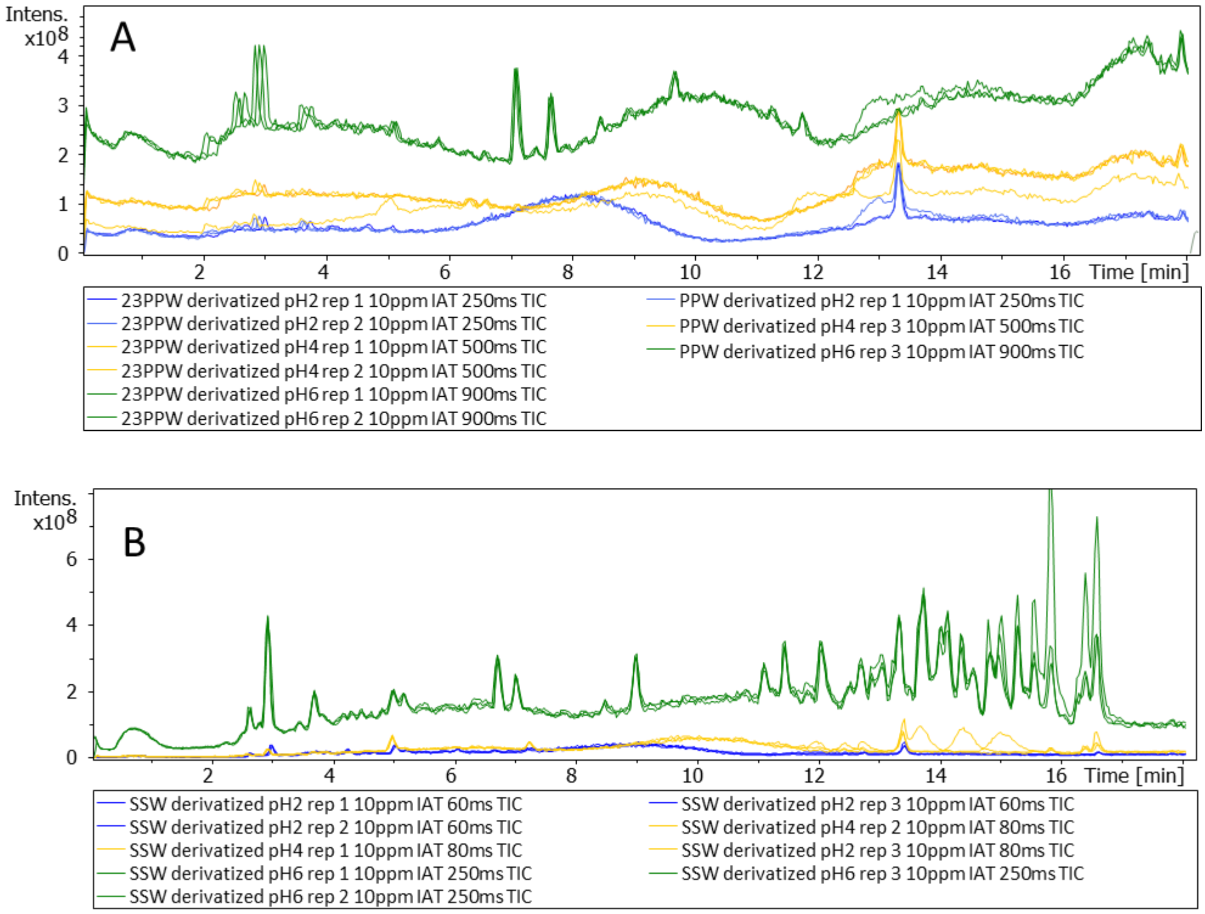

Figure SI 10: Derivatized DOM elution profiles. Total Ion Chromatogram (TICs) for all derivatized samples measured via LC-FT-ICR MS. A PPW and B SSW

5. Electrochemistry

5.1. Details on Electrochemical determination of electron- accepting and -donating capacities

The electron-accepting (EAC) and electron-donating (EDC) capacities of SSPE fractions were measured to quantify the number of quinone functional moieties. For this approach we assume that EAC and EDC in humic substances, as determined by mediated electrochemical reduction (MER) and oxidation (MEO), is mainly provided by quinones.[24] Still, EDC as determined by this technique may partially include phenols due to methodological constraints.[24] Briefly, 200 µg of each fraction was dried under N_2 flow. Under N_2 atmosphere within a glove box (InertLab, Innovative Technology, Amesbury, MA, USA), 250 µL of deionized, degassed water was used to re-dissolve each extract, yielding a concentration of 0.8 mg mL^{-1}. EAC and EDC were determined, as described in Aeschbacher et al. (2010)[24] and Lau et al. (2015)[25], employing a multichannel potentiostat (CH1000, CH Instruments, Austin, TX, USA). Glassy-carbon electrodes (Sigradur® G GAZ 6, HTW GmbH, Thierhaupten, Germany) served as working electrodes. A platinum wire (Sigma Aldrich, St. Louis, USA) encased in a semipermeable glass frit (Glasgerätebau Ochs, Bovenden, Germany) and a silver/silver chloride electrode (Ag/AgCl - RE-1B, ALS Co. Ltd., Tokyo, Japan) were used as counter and reference electrodes, respectively. The electrochemical cells and glass frits were filled with 10 mL and 1.5 mL of a phosphate buffer (pH 7, 0.2 mol L^{-1}) to maintain pH stability, and 0.1 mol L^{-1} KCl was adjusted as background electrolyte. The solutions were continuously stirred. Prior to sample injection, a mediator solution containing either diquat (DQ, 6,7-dihydrodipyrido[1,2-a:2'.1'-c]pyraziniumdibromide monohydrate, E^0h = -0.36 V, Supelco, USA, purity: 99.5%, cDQ = 0.1 mol L^{-1}) for MER or ABTS (2,2-azino-bis-[3-ethylbenzthiazoline-6-sulfonic acid ammonium salt], E^0h = +0.68 V, Sigma Aldrich, St. Louis, USA, purity: 99.5%, cABTS = 0.1 mol L^{-1}) for MEO was added to the cells by removing 180 µL of buffer solution and replacing it with 180 µL of mediator solution. A sample volume of 50 µL, targeting a final amount of approx. 40 µg C per cell, were added to each cell to achieve reproducible peaks of decent size. The reductive or oxidative current response signals in MER and MEO were recorded at 0.2 Hz intervals. All samples were measured in triplicate for EAC determination and once for EDC determination. The current signals were integrated over time and normalized to the amount of carbon added to the cells to obtain the total number of transferred electrons, yielding EAC from MER and EDC from MEO in µmol e- $(g\ C)^{-1}$. We furthermore calculated an oxidation index (OI, eq. 2):

$$\text{Oxidation index (OI)} = \frac{EAC}{(EAC+|EDC|)} \text{ (equation 2)}$$

Higher OI values indicate that redox-sensitive functional groups exist in their oxic form at equilibrium state (N_2 atmosphere), while lower OI values indicate a more reduced state. Note that since extracts were obtained under oxic conditions, the EAC, EDC and OI values to not reflect the redox stat under natural conditions. The Electron exchange capacity was calculated as EEC = EAC+|EDC| an reflects the total number of electron than can be transferred to /from DOM between -0.36V and + 0.68V.

5.2. Results of Electrochemical determination of EAC and EDC

Table SI 5 – Electrochemically determined values for EAC, EDC and calculated oxidation index (OI) for samples (SSW and PPW) extracts at pH 6, 4, and 2.

Source	pH Extracts	EAC (mmol e- / g C)	EDC (mmol e- / g C)	EEC (mmol e- / g C)	Oxidation index (OI)
PPW	6	4.5	-3.4	7.9	0.6
	4	3.5	-3.0	6.5	0.5
	2	8.4	-5.8	14.2	0.6
SSW	6	2.6	-0.9	3.5	0.7
	4	3.0	-0.7	3.7	0.8
	2	3.9	-0.9	4.8	0.8

6. Radiocarbon

6.1. Details on Radiocarbon analysis

Methanol extracts from SSPE fractions were stepwise transferred into tin capsules and dried until the total transferred volume corresponded to 0.3 mg of carbon. The dried residues in the tin capsules were combusted, and the released CO_2 was graphitized. Samples were analyzed alongside modern (oxalic acid) and blank standards using a MICADAS accelerator mass spectrometer (Ionplus, Switzerland) at the Max Planck Institute for Biogeochemistry in Jena.[26] Radiocarbon data were reported as the fraction of modern carbon ($F^{14}C$) (equation 3), calculated as the $^{14}C/^{12}C$ ratio of the sample normalized to a $\delta^{13}C$-value of -25‰, relative to the oxalic acid standard OX-I normalized to a $\delta^{13}C$-value (-19‰); and as an absolute amount of ^{14}C in a sample $\Delta^{14}C$ (in ‰, equation 4) corrected by the radioactive decay of the standard between 1950 and the year of the sample measurement *y*:

$$F^{14}C \frac{\frac{14C}{12C}_{sample,-25}}{0.95\cdot\frac{14C}{12C}_{OX-I,-19}} \text{ (equation 3)}$$

$$\Delta^{14}C = \left(\frac{\frac{14C}{12C}_{sample,-25}}{0.95\cdot\frac{14C}{12C}_{OX-I,-19}\cdot e^{(y-1950)/8267}} - 1\right)\cdot 1000 \text{ (equation 4)}$$

6.2. Results on Radio carbon analysis

Table SI 6 – Radiocarbon data for PPW and SSW

	PPW				SSW			
	$F^{14}C$	err	$\Delta^{14}C$ (‰)	err (‰)	$F^{14}C$	err	$\Delta^{14}C$ (‰)	err (‰)
pH 6	0.8694	0.0032	-138.4	3.2				
pH 4	0.8421	0.0032	-165.4	3.2	0.8009	0.0032	-206.2	3.2
pH 2	0.8510	0.0031	-156.5	3.1	0.7917	0.0032	-215.4	3.2

7. Results and discussion

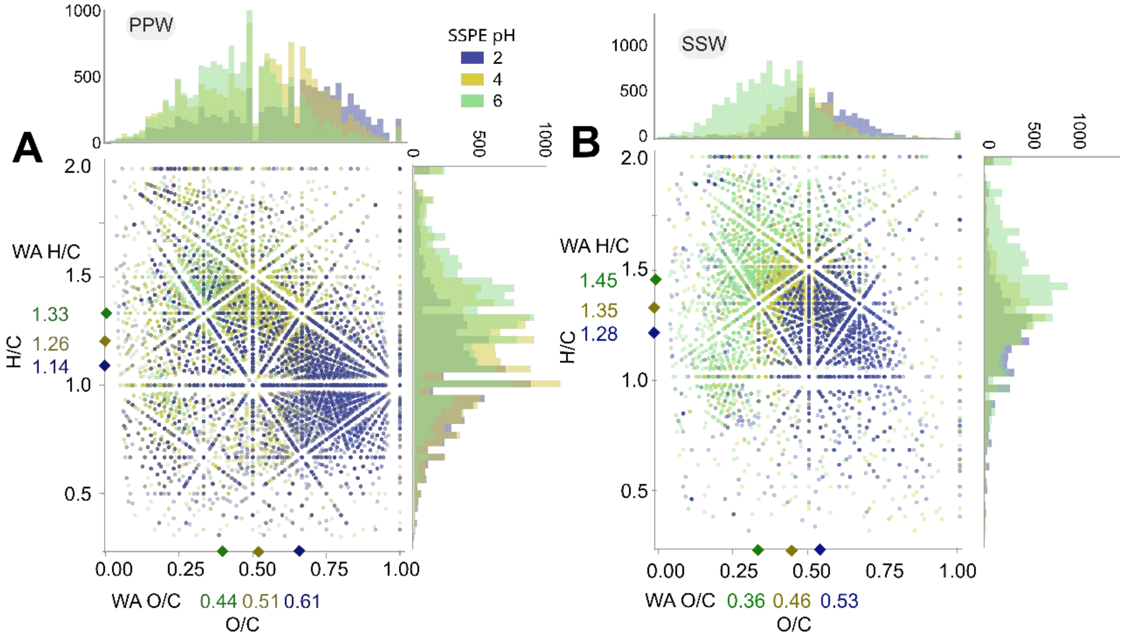

Figure SI 11 – Van Krevelen diagram of MFs found in pH 2 (blue), pH 4 (yellow) and pH 6 (green) for PPW (left) and SSW (right). The marginal histograms represent the count of MFs. The weight average (WA) are depicted with diamonds at the respective SSPE (pH) color. All the segments of each extract were summed.

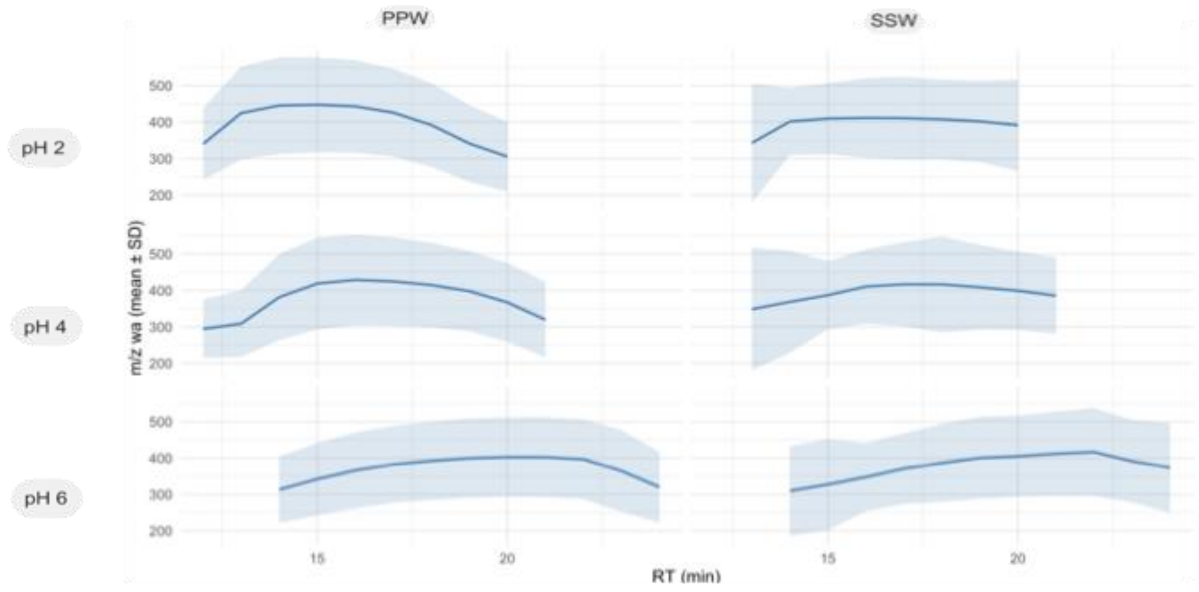

Figure SI 12 – Weighted average distribution of the m/z (m/z_{wa}) against RT (min) of SSW (right) and PPW (left) for sequential solid-phase extraction (SSPE) at pH 2, pH 4 and pH 6.

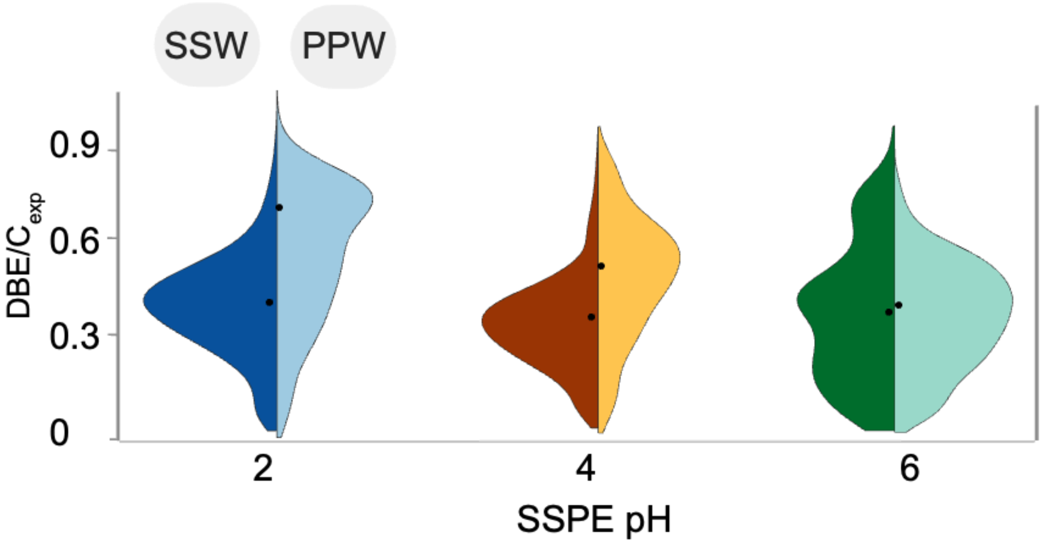

Figure SI 13 – Double bond equivalent to carbon ratio (DBE/C_{exp}) distribution of SSW (left, darker colors) and PPW (right, light colors) for sequential solid-phase extraction (SSPE) at pH 2 (blue), pH 4 (yellow) and pH 6 (green). The dotted lines indicate the modes i.e. most repeated values of dispersity indices.

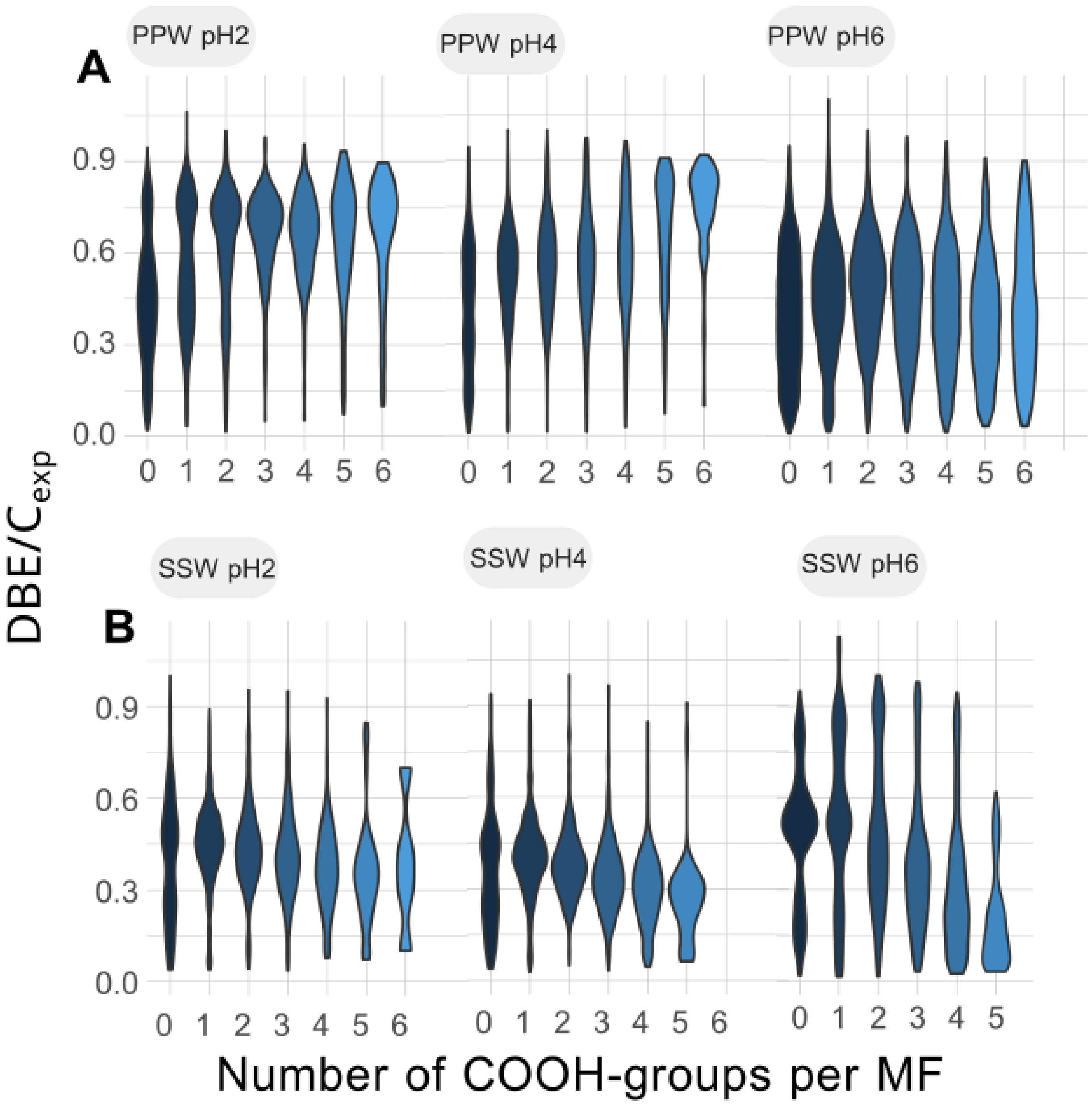

Figure SI 14 – Double bond equivalent to carbon ratio (DBE/C_{exp}) distribution of number of COOH-groups per MF for PPW (A) and SSW (B) colored by COOH-group

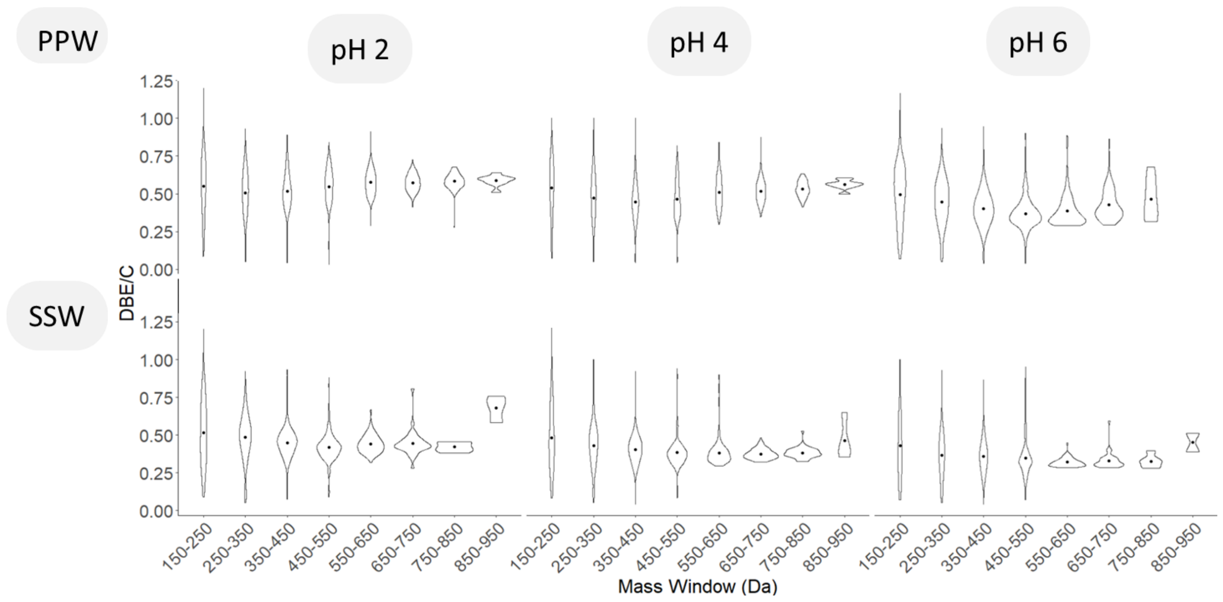

Figure SI 15 - Distribution of DBE/C across different mass windows for each source and group. The upper panel represents PPW, while the lower panel represents SSW. The mean values are indicated by black dots.

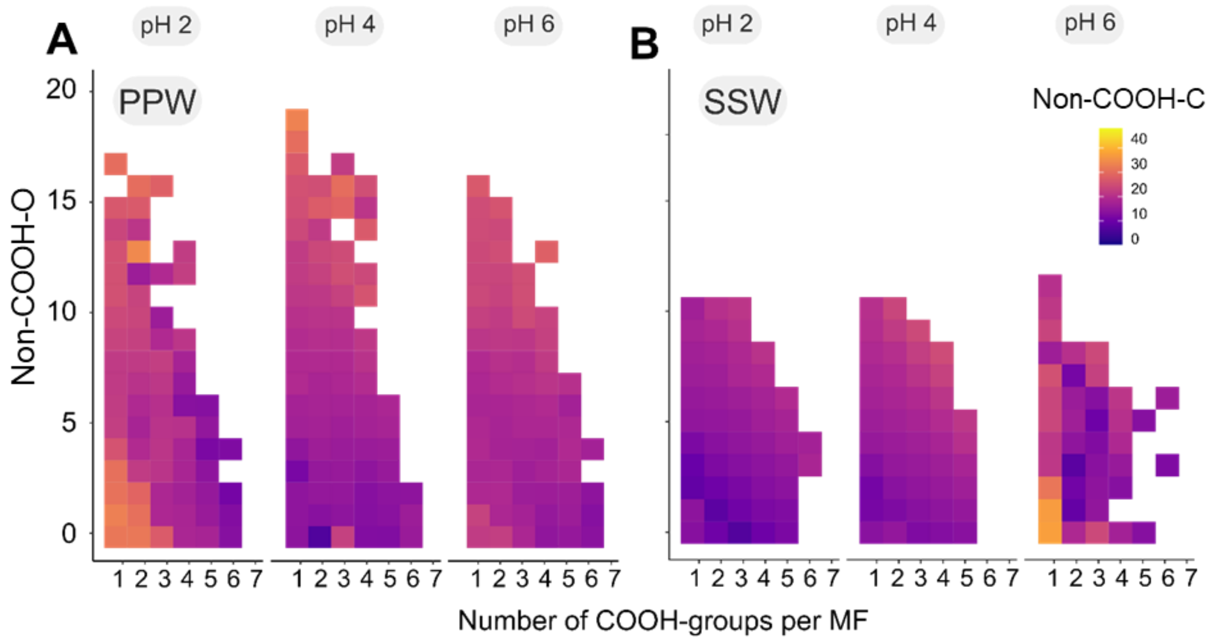

Figure SI 16 - Distribution of oxygens atoms not bounded as carboxylic acid groups (non-COOH-O) against the number of COOH obtained experimentally by derivatization. The heat map is colored by the number of carbon atoms not bound in COOH-groups (non-COOH-C). In the pH 6 fraction of SSW, an increasing number of non-COOH-C per MF was observed, which was supported by the increase in alkyl functionalities seen in the 1H NMR (Figure 1 D). Together with the overall low number of COOH-groups at pH 6, this suggests that these

compounds were predominantly fatty acids and wax-related carotenoids—expected degradation products of aquatic primary producers in surface seawater.[27,28]

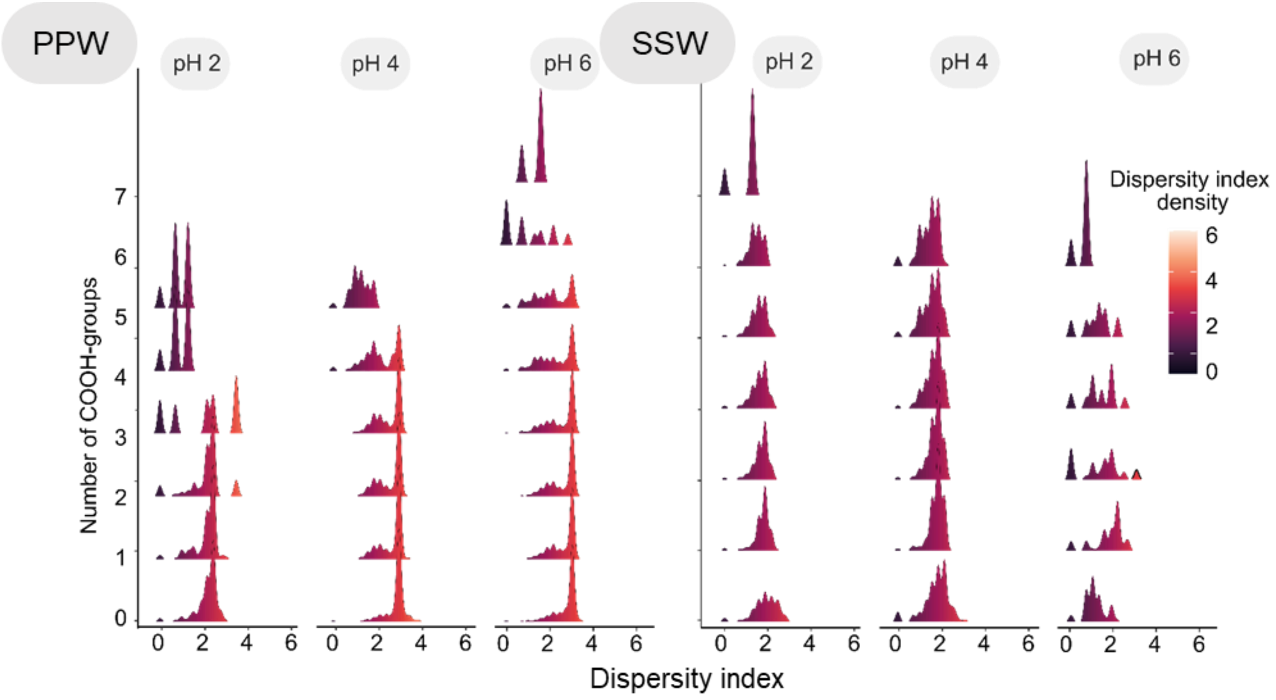

Figure SI 17 – Density of dispersity indexes for each COOH-group. PPW can be found on the left and SSW on the right. Peak at dispersity index 3.02 (pH 4 and 6) indicates high frequency of MFs with COOH-groups for PPW.

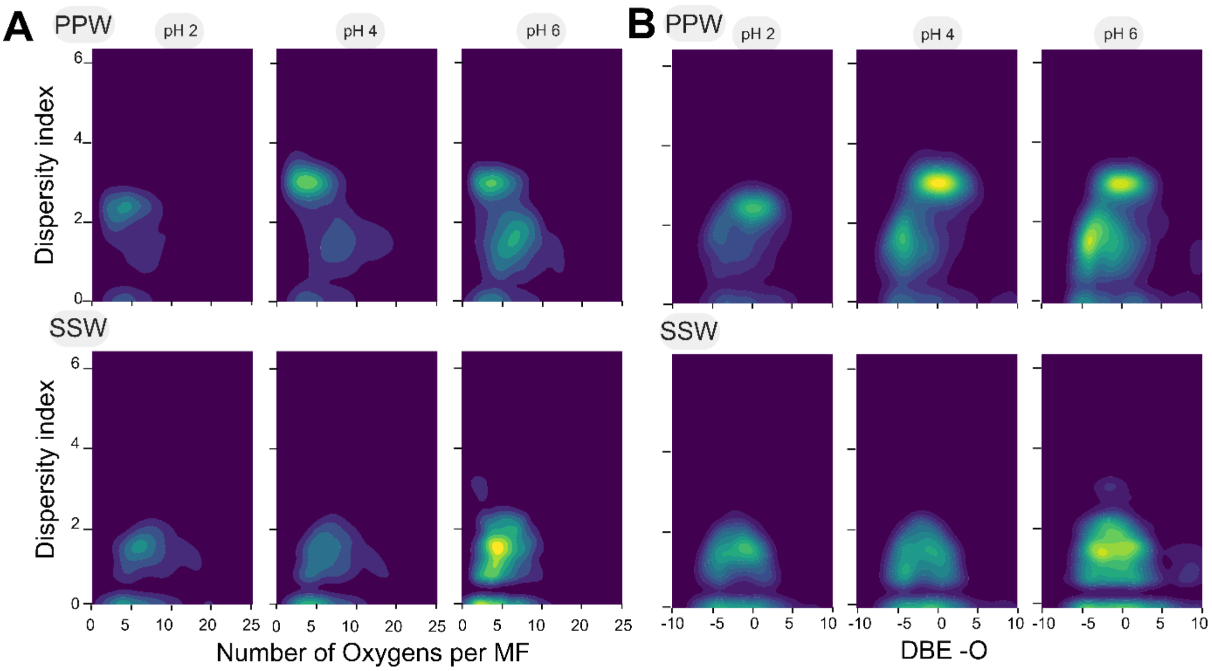

Figure SI 18 – Dispersity indexes density plotted against the **a.** number of O and **b**. DBE-O. The upper row represents the SSW while the bottom PPW. The left column is pH 2, the second pH 4 and the third pH 6.

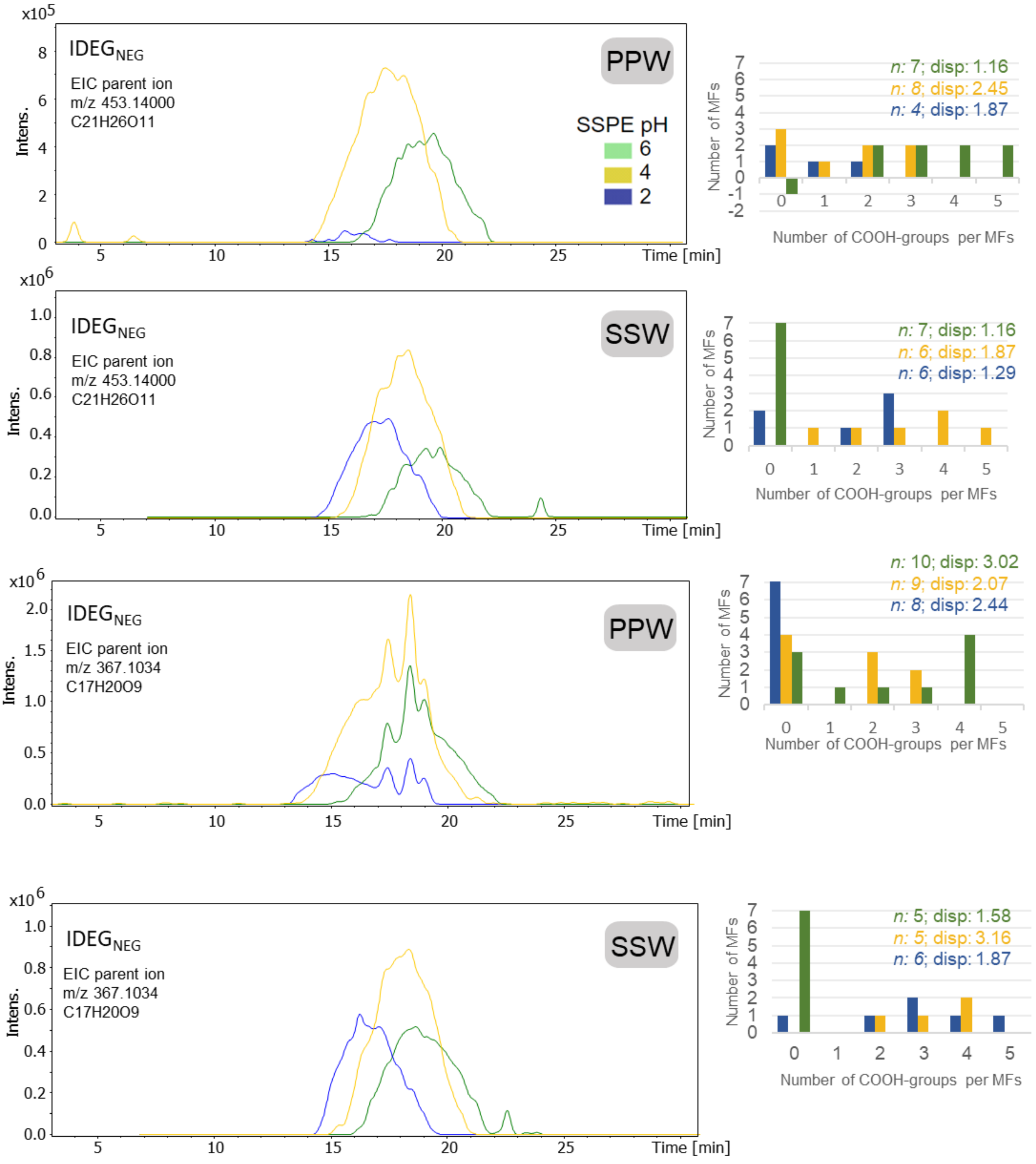

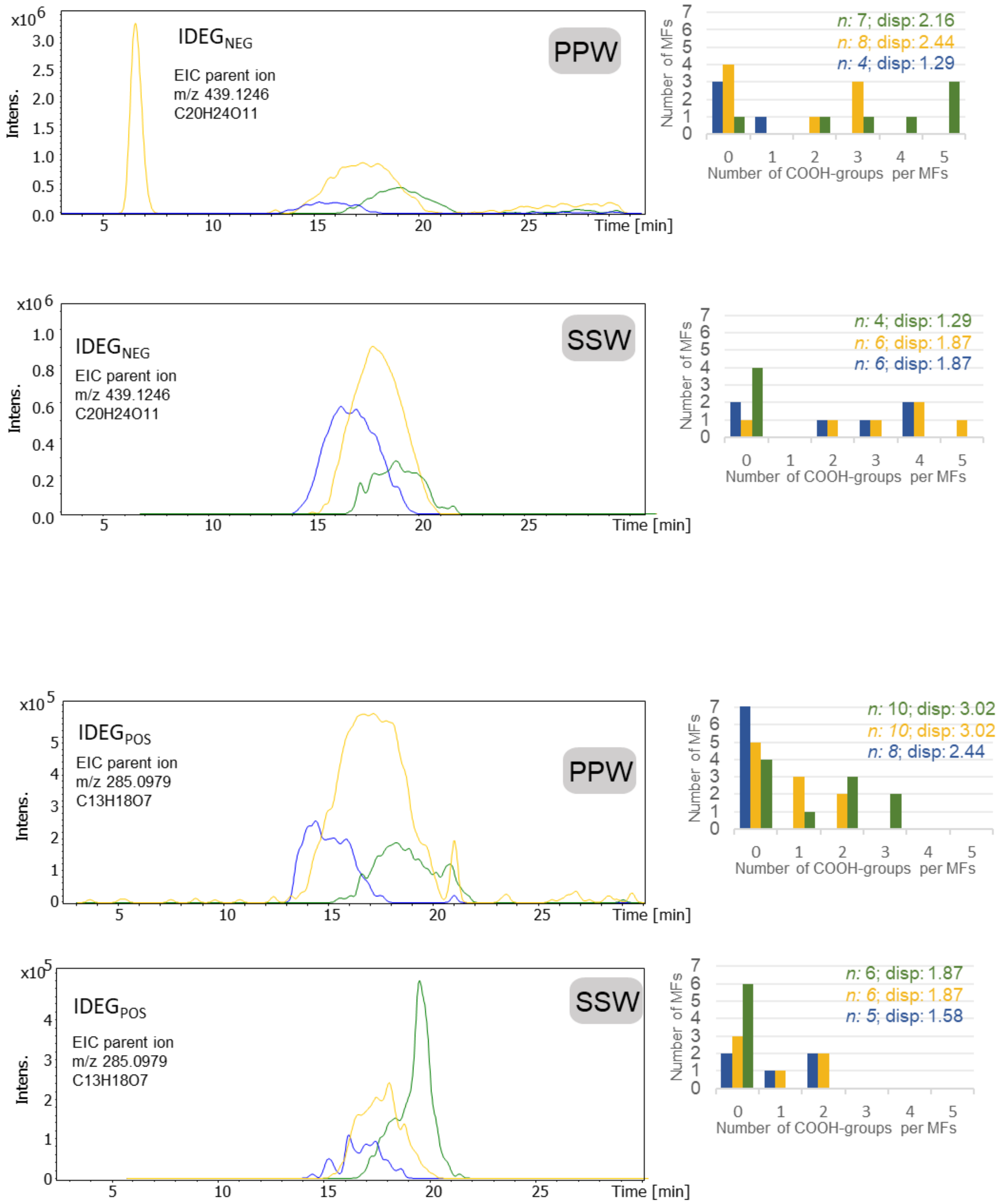

x10^6
IDEG_NEG
EIC parent ion
m/z 439.1246
C20H24O11
PPW
Intens.
Time [min]
n: 7; disp: 2.16
n: 8; disp: 2.44
n: 4; disp: 1.29
Number of MFs
Number of COOH-groups per MFs
SSW
n: 4; disp: 1.29
n: 6; disp: 1.87
n: 6; disp: 1.87
x10^5
IDEG_POS
EIC parent ion
m/z 285.0979
C13H18O7
n: 10; disp: 3.02
n: 10; disp: 3.02
n: 8; disp: 2.44
n: 6; disp: 1.87
n: 6; disp: 1.87
n: 5; disp: 1.58

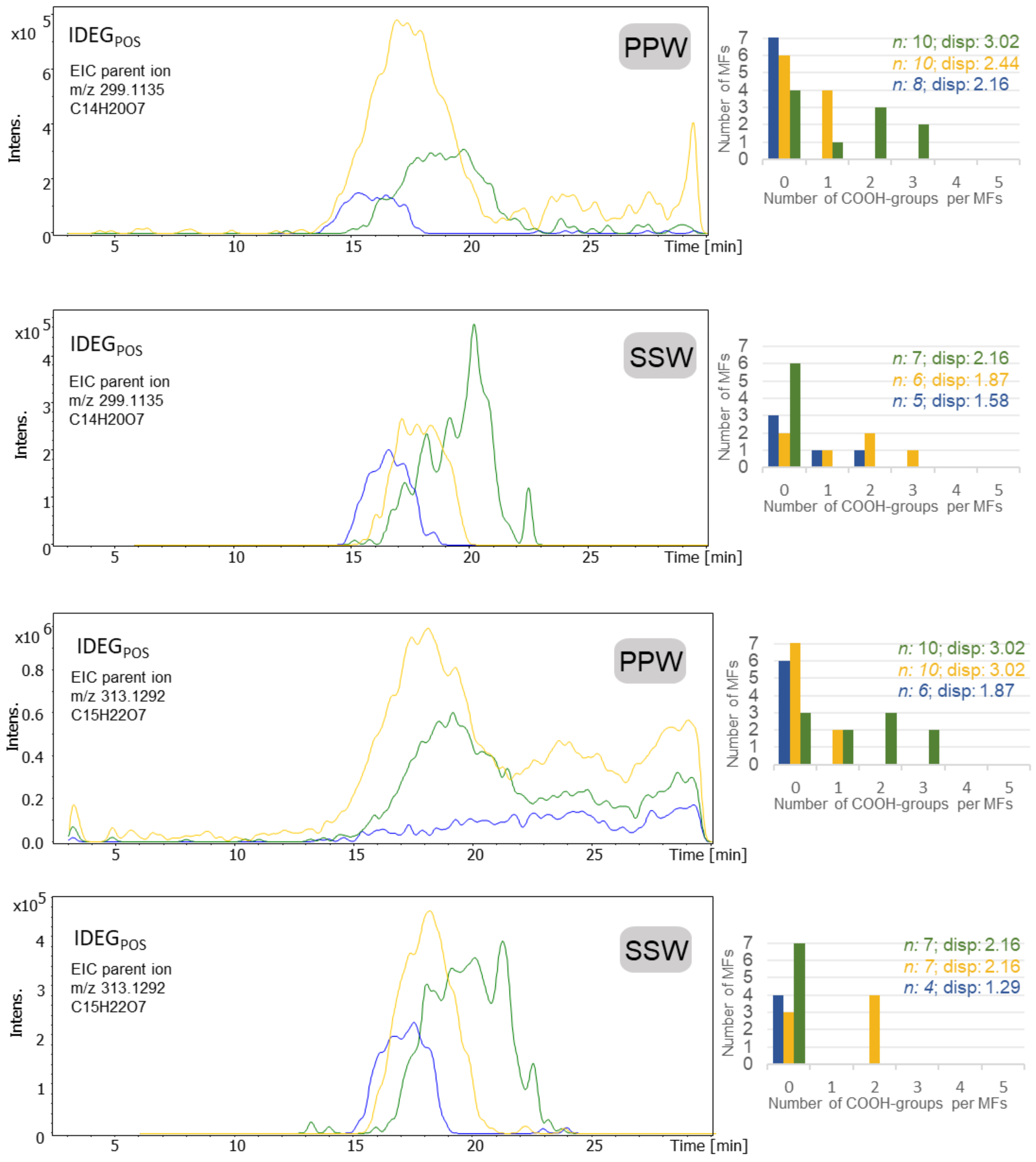
x10^5
IDEG POS
EIC parent ion
m/z 299.1135
C14H20O7
PPW
Intens.
Time [min]
n: 10; disp: 3.02
n: 10; disp: 2.44
n: 8; disp: 2.16
Number of MFs
Number of COOH-groups per MFs
x10^5
IDEG POS
EIC parent ion
m/z 299.1135
C14H20O7
SSW
Intens.
Time [min]
n: 7; disp: 2.16
n: 6; disp: 1.87
n: 5; disp: 1.58
Number of MFs
Number of COOH-groups per MFs
x10^6
IDEG POS
EIC parent ion
m/z 313.1292
C15H22O7
PPW
Intens.
Time [min]
n: 10; disp: 3.02
n: 10; disp: 3.02
n: 6; disp: 1.87
Number of MFs
Number of COOH-groups per MFs
x10^5
IDEG POS
EIC parent ion
m/z 313.1292
C15H22O7
SSW
Intens.
Time [min]
n: 7; disp: 2.16
n: 7; disp: 2.16
n: 4; disp: 1.29
Number of MFs
Number of COOH-groups per MFs

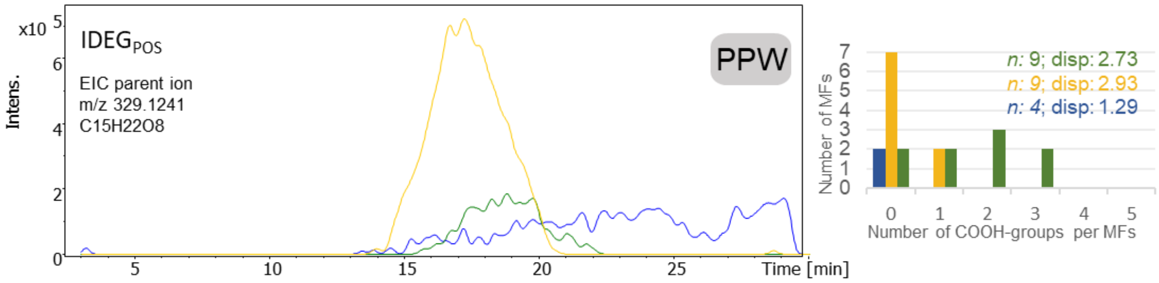
x10 5
Intens.
IDEGPOS
EIC parent ion
m/z 329.1241
C15H22O8
PPW
Time [min]
Number of MFs
Number of COOH-groups per MFs
n: 9; disp: 2.73
n: 9; disp: 2.93
n: 4; disp: 1.29

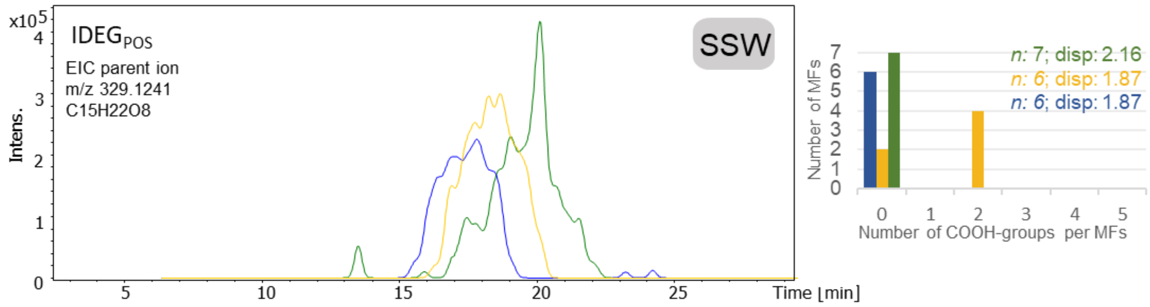
x10 5
Intens.
IDEGPOS
EIC parent ion
m/z 329.1241
C15H22O8
SSW
Time [min]
Number of MFs
Number of COOH-groups per MFs
n: 7; disp: 2.16
n: 6; disp: 1.87
n: 6; disp: 1.87

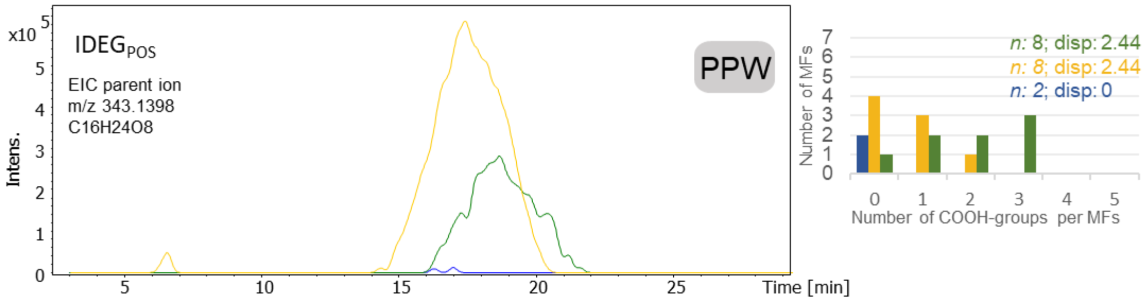
x10 5
Intens.
IDEGPOS
EIC parent ion
m/z 343.1398
C16H24O8
PPW
Time [min]
Number of MFs
Number of COOH-groups per MFs
n: 8; disp: 2.44
n: 8; disp: 2.44
n: 2; disp: 0

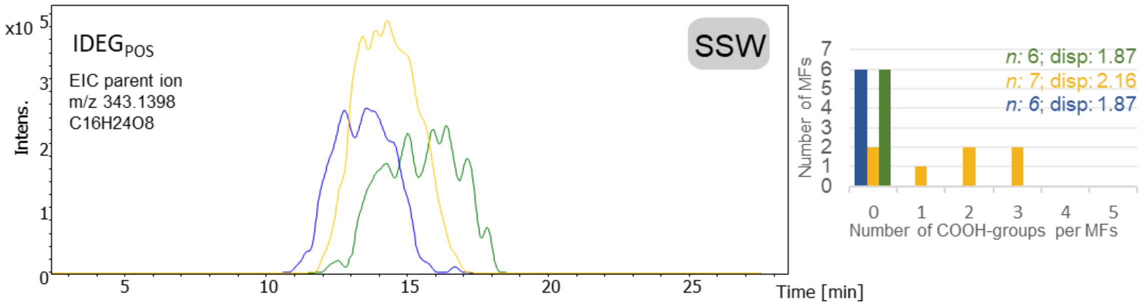
x10 5
Intens.
IDEGPOS
EIC parent ion
m/z 343.1398
C16H24O8
SSW
Time [min]
Number of MFs
Number of COOH-groups per MFs
n: 6; disp: 1.87
n: 7; disp: 2.16
n: 6; disp: 1.87

Figure SI 19 – Structural isomeric information on negative (IDEGNEG and positive (IDEGPOS) degradation indices. Extracted ion chromatograms (EICs) are shown for PPW (top) and SSW (bottom) at pH levels of 6 (green), 4 (yellow), and 2 (blue). The bar plots represent the number of carboxylic acid groups (COOH-groups) found *per* molecular formula (MF), as determined by derivatization. "n" indicates the number of isomers found at each pH, and "disp." refers to their dispersity index. Number of MFs "-1" means there is one more isomer detected in the labeled samples compared to the total number of MF in the non-labeled extract. The ten I_{DEG} MF (5 NEG_{IDEG} and 5 POS_{IDEG}) are consistently detected across DOM samples. I_{DEG} is an empirical proxy for the relative age or degradation state of an individual sample within a given set of samples. Concerning their molecular composition, NEG_{IDEG} MF have a lower hydrogen-to-carbon (H/C) ratio compared to POS_{IDEG} MF (but no difference in O/C ratios) and are part of the MF that were suggested to represent CRAM.[29,30]

References

1. Blodau, C. Carbon cycling in peatlands - A review of processes and controls. *Environmental Reviews* **134**, 111–134 (2002).
2. Hertkorn, N., Harir, M., Koch, B. P., Michalke, B. & Schmitt-Kopplin, P. High-field NMR spectroscopy and FTICR mass spectrometry: Powerful discovery tools for the molecular level characterization of marine dissolved organic matter. *Biogeosciences* **10**, 1583–1624 (2013).
3. Matos, R. R. *et al.* Post column infusion of an internal standard into LC-FT-ICR MS enables semi-quantitative comparison of dissolved organic matter in original samples. *Analyst* **149**, 3468–3478 (2024).
4. Wurz, J., Groß, A., Franze, K. & Lechtenfeld, O. Lambda-Miner: Enhancing Reproducible Natural Organic Matter Data Processing with a Semi-Automatic Web Application *EGU General Assembly 2024, Vienna, Austria* **EGU24-15782**, (2024).
5. Herzsprung, P. *et al.* Understanding molecular formula assignment of Fourier transform ion cyclotron resonance mass spectrometry data of natural organic matter from a chemical point of view. *Anal Bioanal Chem* **406**, 7977–7987 (2014).
6. Gao, S. *et al.* Detection and Exclusion of False-Positive Molecular Formula Assignments via Mass Error Distributions in UHR Mass Spectra of Natural Organic Matter. *Anal Chem* **96**, 10210–10218 (2024).

7. Han, L., Kaesler, J., Peng, C., Reemtsma, T. & Lechtenfeld, O. J. Online counter gradient LC-FT-ICR-MS enables detection of highly polar natural organic matter fractions. *Anal Chem* **93**, 1740–1748 (2021).

8. Dittmar, T., Koch, B., Hertkorn, N. & Kattner, G. A simple and efficient method for the solid-phase extraction of dissolved organic matter (SPE-DOM) from seawater. *Limnol Oceanogr Methods* **6**, 230–235 (2008).

9. Matos, R. R. *et al.* Post column infusion of an internal standard into LC-FT-ICR MS enables semi-quantitative comparison of dissolved organic matter in original samples. *Analyst* **149**, 3468–3478 (2024).

10. Bray, J. R. & Curtis, J. T. An ordination of the upland forest communities of southern Wisconsin. *Ecol Monogr* **27**, 325–347 (1957).

11. Kong, X., Jendrossek, T., Ludwichowski, K. U., Marx, U. & Koch, B. P. Solid-Phase Extraction of Aquatic Organic Matter: Loading-Dependent Chemical Fractionation and Self-Assembly. *Environ Sci Technol* **55**, 15495–15504 (2021).

12. Lechtenfeld, O. J., Kaesler, J., Jennings, E. K. & Koch, B. P. Direct Analysis of Marine Dissolved Organic Matter Using LC-FT-ICR MS. *Environ Sci Technol* https://doi.org/10.1021/acs.est.3c07219 (2024) doi:10.1021/acs.est.3c07219.

13. Hertkorn, N., Harir, M., Koch, B. P., Michalke, B. & Schmitt-Kopplin, P. High-field NMR spectroscopy and FTICR mass spectrometry: Powerful discovery tools for the molecular level characterization of marine dissolved organic matter. *Biogeosciences* **10**, 1583–1624 (2013).

14. Jennings, E. K. Investigating Effluent Organic Matter Using Novel Ozonation and LC-FT-ICR-MS. (2023).

15. Han, L., Kaesler, J., Peng, C., Reemtsma, T. & Lechtenfeld, O. J. Online counter gradient LC-FT-ICR-MS enables detection of highly polar natural organic matter fractions. *Anal Chem* **93**, 1740–1748 (2021).

16. Felgate, S. L., Craig, A. J., Moodie, L. W. K. & Hawkes, J. Characterization of a Newly Available Coastal Marine Dissolved Organic Matter Reference Material (TRM-0522). https://doi.org/10.1021/acs.analchem.2c05304 (2023) doi:10.1021/acs.analchem.2c05304.

17. Alexander J. Craig, Lindon W. K. Moodie, and J. A. H. The Synthesis of Carboxylate Rich Alicyclic Molecules for the Investigation of the Chemical Space of Dissolved Organic Matter. (2023).

18. Zherebker, A. *et al.* Enumeration of carboxyl groups carried on individual components of humic systems using deuteromethylation and Fourier transform mass spectrometry. *Anal Bioanal Chem* **409**, 2477–2488 (2017).

19. Zherebker, A. *et al.* Refinement of Compound Aromaticity in Complex Organic Mixtures by Stable Isotope Label Assisted Ultrahigh-Resolution Mass Spectrometry. *Anal Chem* **92**, 9032–9038 (2020).

20. Zherebker, A. *et al.* Optical Properties of Soil Dissolved Organic Matter Are Related to Acidic Functions of Its Components as Revealed by Fractionation, Selective Deuteromethylation, and Ultrahigh Resolution Mass Spectrometry. *Environ Sci Technol* **54**, 2667–2677 (2020).

21. Zherebker, A., Rukhovich, G. D., Sarycheva, A., Lechtenfeld, O. J. & Nikolaev, E. N. Aromaticity Index with Improved Estimation of Carboxyl Group Contribution for Biogeochemical Studies. *Environ Sci Technol* **56**, 2729−2737 (2022).

22. Witt, M., Fuchser, J. & Koch, B. P. Fragmentation studies of fulvic acids using collision induced dissociation fourier transform ion cyclotron resonance mass spectrometry. *Anal Chem* **81**, 2688–2694 (2009).

23. Plancque, G., Amekraz, B., Moulin, V., Toulhoat, P. & Moulin, C. Molecular structure of fulvic acids by electrospray with quadrupole time-of-flight mass spectrometry. *Rapid Communications in Mass Spectrometry* **15**, 827–835 (2001).

24. Aeschbacher, M., Graf, C., Schwarzenbach, R. P. & Sander, M. Antioxidant properties of humic substances. *Environ Sci Technol* **46**, 4916–4925 (2012).

25. Lau, M. P., Sander, M., Gelbrecht, J. & Hupfer, M. Solid phases as important electron acceptors in freshwater organic sediments. *Biogeochemistry* **123**, 49–61 (2015).

26. Steinhof, A. Data Analysis at the Jena 14 C Laboratory . *Radiocarbon* **55**, 282–293 (2013).

27. Koch, B. P. & Dittmar, T. From mass to structure: An aromaticity index for high-resolution mass data of natural organic matter. *Rapid Communications in Mass Spectrometry* **20**, 926–932 (2006).

28. Zherebker, A., Rukhovich, G. D., Sarycheva, A., Lechtenfeld, O. J. & Nikolaev, E. N. Aromaticity Index with Improved Estimation of Carboxyl Group Contribution for Biogeochemical Studies. *Environ Sci Technol* **56**, 2729−2737 (2022).

29. Flerus, R. *et al.* A molecular perspective on the ageing of marine dissolved organic matter. *Biogeosciences* **2**, 1935–1955 (2012).

30. Lechtenfeld, O. J. *et al.* Molecular transformation and degradation of refractory dissolved organic matter in the Atlantic and Southern Ocean. *Geochim Cosmochim Acta* **126**, 321–337 (2014).
